# Monoclonal Antibodies Accessing the Cytosol of Living Cells and Binding to Polo‐Like Kinase 1 Interdomain Linker Affect Mitotic Behavior

**DOI:** 10.1002/cbic.202500858

**Published:** 2026-04-29

**Authors:** Clément Steyer, Jean-Christophe Amé, Mariel Donzeau, Karie Shen, Manuela Chiper, Guy Zuber

**Affiliations:** ^1^ UMR 7242, Biotechnology and Cell Signaling Université de Strasbourg CNRS Illkirch France

**Keywords:** cytosolic delivery, electroporation, monoclonal antibodies, polo-like kinase 1

## Abstract

Polo‐like kinase 1 (PLK1) is a master regulator of mitosis and a well‐established driver of cancer progression. Strategies to target PLK1 have largely focused on RNA interference using small interfering RNAs or small‐molecule inhibitors. Here, we introduce the direct intracellular targeting of the PLK1 interdomain linking the polo‐box domain to the kinase domain using monoclonal antibodies. Three monoclonal antibodies recognizing the interdomain region were delivered into the cytosol of HeLa cells by electroporation. These antibodies were shown to bind PLK1 in living cells and to modulate mechanisms involved in mitotic progression. Our results establish these antibodies as tools to probe PLK1 function through real‐time labeling and interference with the mitotic machinery.

## Graphical abstract

Monoclonal antibodies targeting the interdomain region of polo‐like kinase 1 (PLK1) were delivered into living HeLa cells by electroporation to directly modulate mitotic progression. This intracellular antibody approach provides a new tool to probe PLK1 function and highlights the potential of antibodies to regulate key mitotic regulators inside cells.

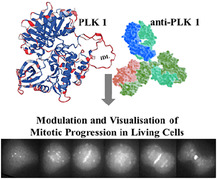

## 1 Introduction

**FIGURE 1:**
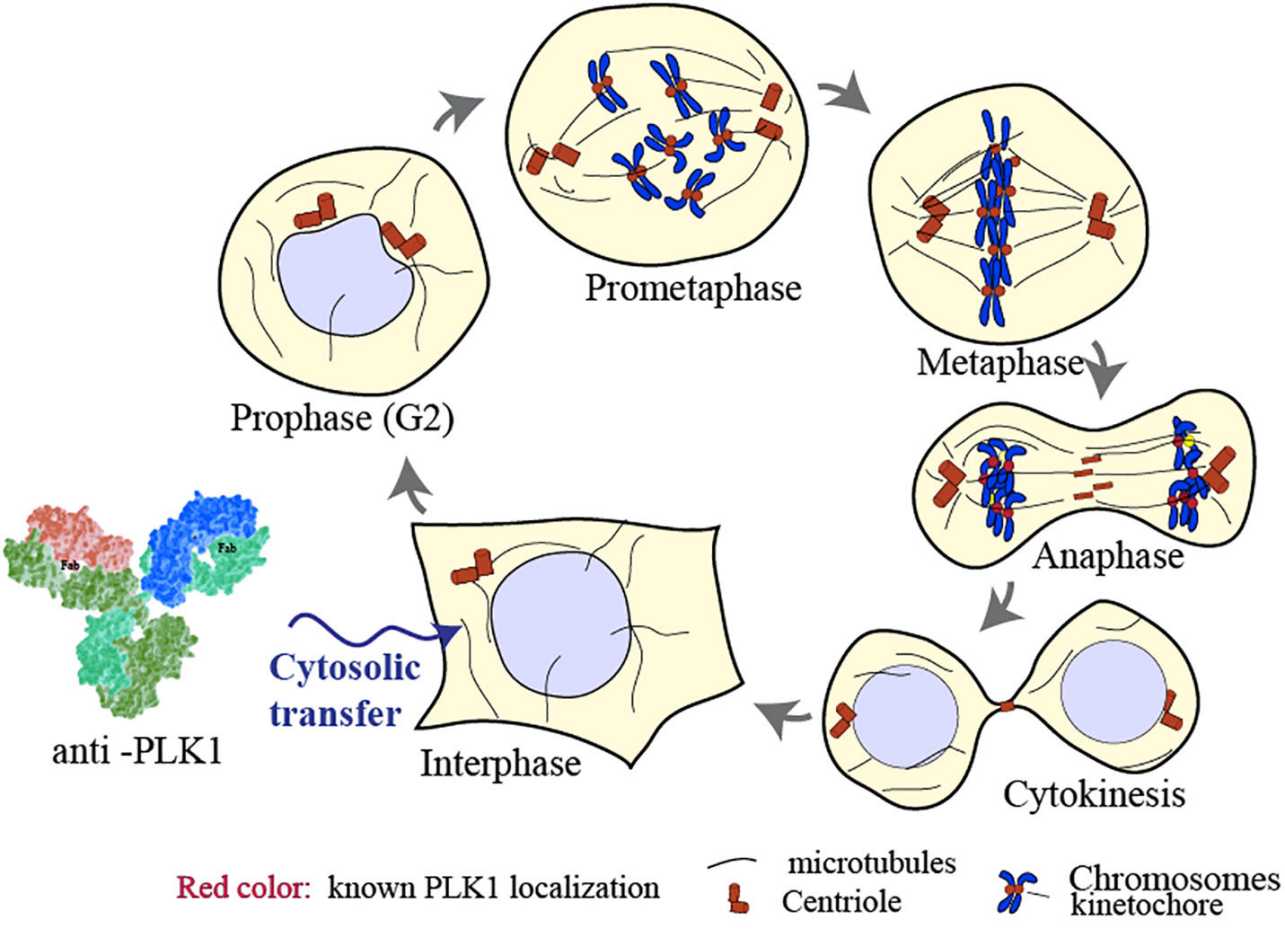
The mitotic regulator PLK1 is a master controller of the mitotic progression and localizes to key sites during this event. mAbs were delivered into living cells via transient plasma membrane permeabilization using nonlethal electric pulses to evaluate selective binding in native conditions and modulation of the target function.

Mammalian cell division relies on precise spatiotemporal control of mitotic regulators, with polo‐like kinase 1 (PLK1) [1] acting as a master orchestrator [2, 3, 4]. PLK1 undergoes phosphorylation and localizes to centrosomes, kinetochores, the spindle midzone, and the midbody during mitosis (Figure 1) [5]. PLK1 expression is tightly regulated: transcripts peak at S/G2 transition [1], kinase activation occurs then at G2/M during mitosis [3, 6]. Protein levels remain low in most adult tissues [7]. In cancer, however, overexpression of PLK1 [8, 9, 10] and phosphorylation at Thr210 [11] correlates with aggressive disease and poor prognosis. This dual role as biomarker and driver makes PLK1 an attractive therapeutic target [12, 13, 14]. Efforts to block PLK1 kinase activity have yielded potent ATP‐competitive inhibitors such as BI 2536 and BI 6727 (Volasertib), which display sub‐nanomolar potency and high selectivity. Both compounds inhibit tumor growth in vitro. Volasertib advanced to clinical trials owing to favorable pharmacokinetics [2, 15], although its efficacy in acute myeloid leukemia patients remained limited [16, 17, 18]. Onvansertib, a highly selective next‐generation inhibitor, has shown robust antitumor activity [1, 19, 20] and has also undergone clinical evaluation [2, 3, 4, 5, 14, 21]. Yet, resistance and toxicity associated with kinase inhibitors highlight the need for a deeper understanding of the cellular machinery regulated by PLK1 and for alternative strategies to target this kinase. Targeting the polo‐box domain (PBD) represents one such approach. Poloxin [5, 22, 23], phosphopeptides [1, 24, 25, 26], and triazoloquinazolinones [3, 6, 27] disrupt PBD function and impair cancer cell proliferation. Remarkably, as early as 1996, cytoplasmic injection of polyclonal anti‐PLK1 antibodies was shown to inhibit mitosis [7, 28], establishing a proof‐of‐concept for antibody‐mediated interference. Advances in antibody engineering [29, 30, 31] and intracellular delivery technologies [32, 33, 34], prompt a reassessment of targeting PLK1 in cells with monoclonal antibodies.

Here, we further characterized three commercially available anti‐PLK1 monoclonal antibodies and then evaluated their PLK1 targeting ability in living HeLa cells using electroporation (Figure 1). Our results provide evidence that antibodies targeting the interdomain of PLK1 have dual activities: they can dynamically probe the localization of PLK1 in living cells while perturbing mitotic progression when the intracellular antibody to PLK1 ratio approaches equimolarity [35].

## 2 Results and Discussion

**FIGURE 2:**
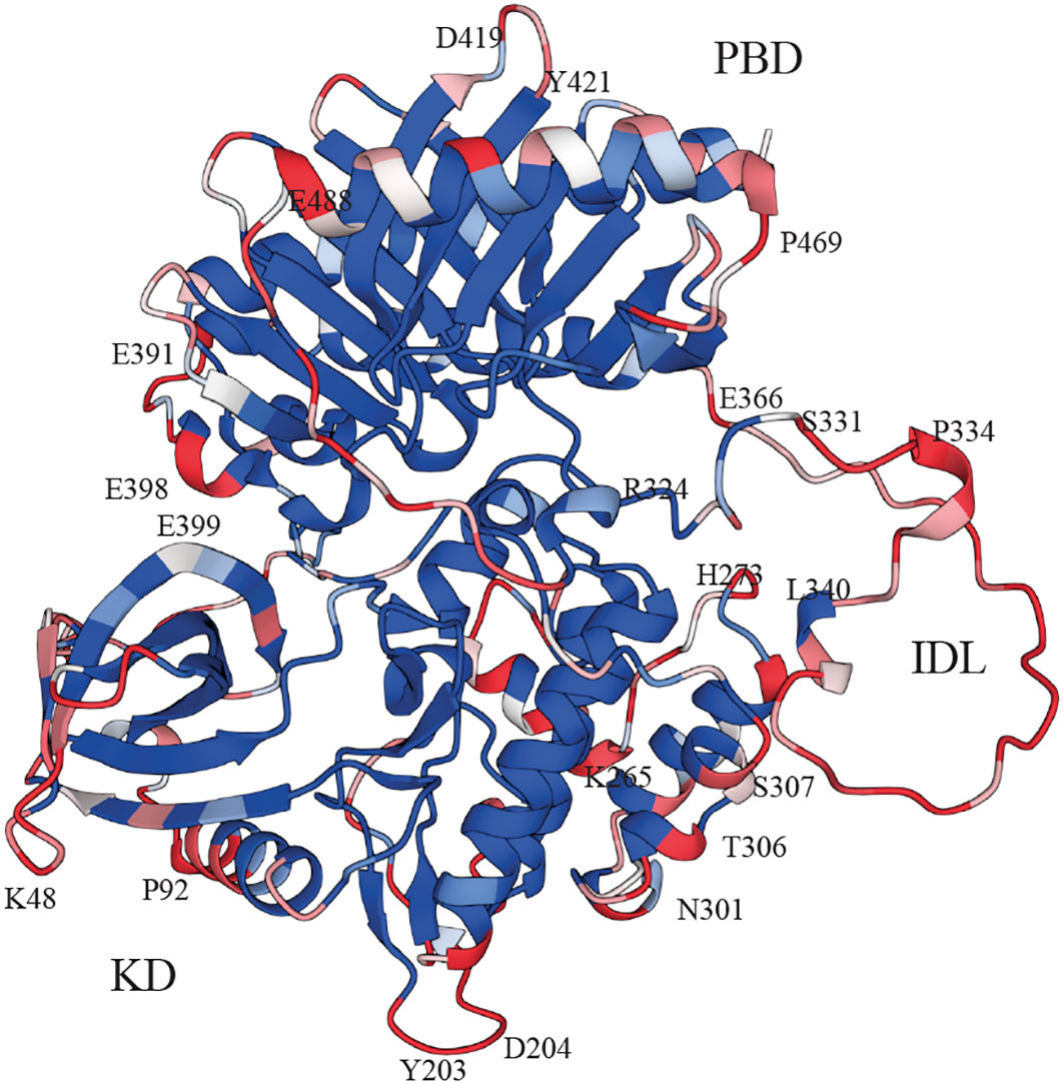
Predicted human PLK1 structure showing secondary folds and B‐cell epitope propensity (red gradient). Structure (UniProt P53350) was predicted by AlphaFold; B‐cell epitope propensity was analyzed with DiscoTope 3.0.

Human PLK1 is a 603‐amino‐acid protein composed of a highly conserved N‐terminal serine/threonine kinase domain (KD, residues 1–305) and a C‐terminal polo‐box domain (PBD, residues 410–603) connected by an interdomain linker (IDL, residues 306–409). The crystal structures of the KD (PDB ID: 2OU7) [8, 9, 10, 36] and the PBD (PDB ID: 5NFU) [11, 16, 18] were solved at resolutions below 2.4 Å. The complete structure, including the flexible IDL, was predicted using AlphaFold database (https://alphafold.ebi.ac.uk/search/text/P53350) and used here to estimate B‐cell epitope propensity with DiscoTope‐3 (Figure 2) [12, 13, 14, 19] Predicted epitopes were scattered across the KD and PBD, but the IDL segment (residues 331–366) appeared as a hotspot for antibody recognition, likely reflecting its flexibility and surface accessibility.

**FIGURE 3:**
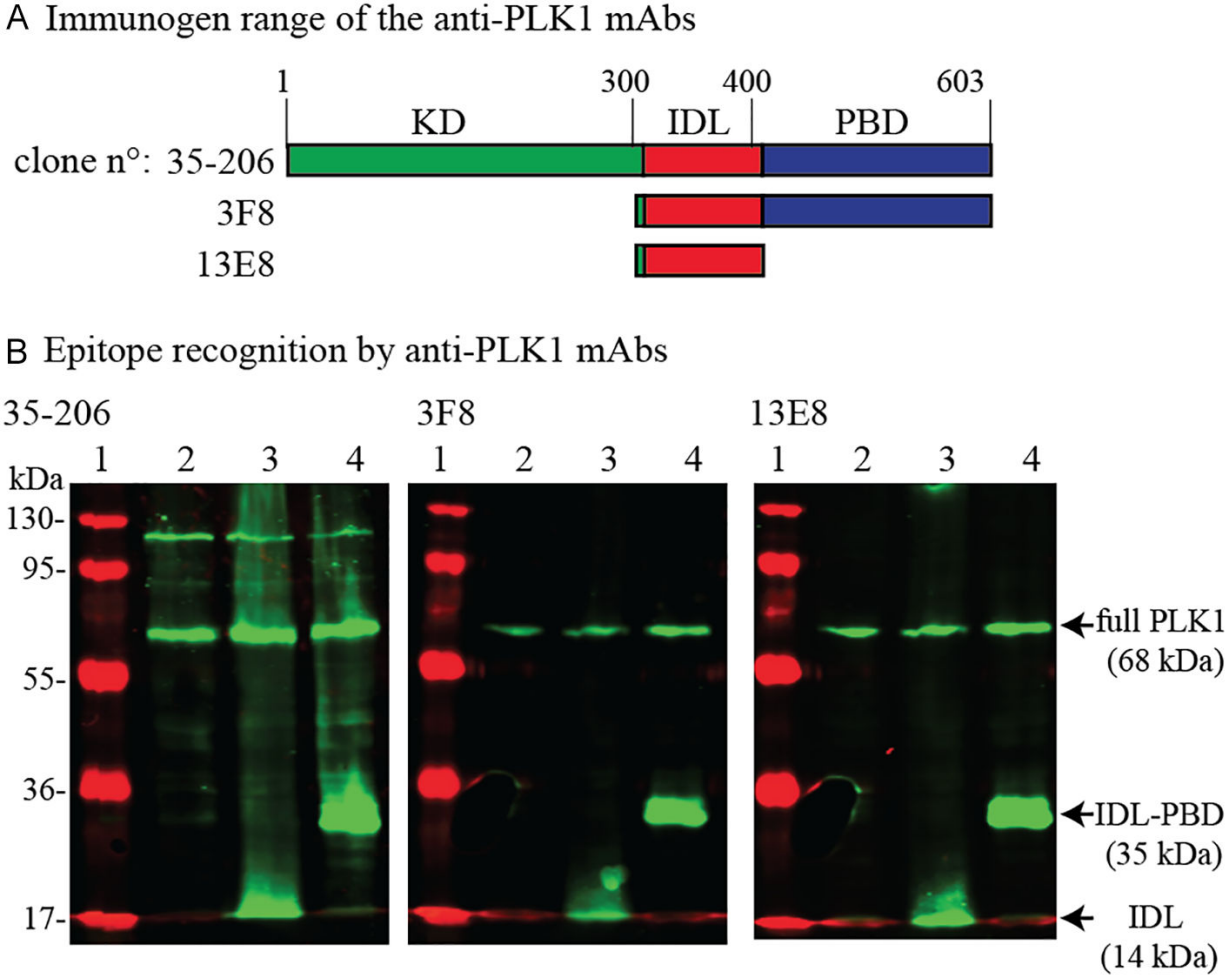
Anti‐PLK1 mAbs characterization. (A) Clones 35‐206, 3F8, and 13E8 were raised against full‐length PLK1, fragment 300–600, or fragment 300–400, respectively. KD: Kinase Domain; IDL: Interdomain Linker; PBD: Polo Box Domain. (B) Epitope mapping by Western blot. Lane 1: protein ladder; Lanes 2‐4: U2OS cells expressing 68 kDa PLK1, 14 kDa IDL (300–400), or 35 kDa IDL‐PBD (300–603), respectively.

We then examined three commercially available monoclonal antibodies against PLK1 (clones 35–206, 3F8, and 13E8), raised against the full‐length protein (603 aa, 68 kDa), the C‐terminal fragment spanning residues 300–603 (IDL–PBD, 35 kDa), and the IDL fragment 300–400 (14 kDa), respectively (Figure 3A). Western blotting with extracts of U2OS cells expressing native PLK1, the 35 kDa IDL–PBD fragment, or the 14 kDa IDL fragment revealed that all three antibodies recognize the IDL (Figure 3B). Clones 3F8 and 13E8 appeared specific for PLK1, whereas clone 35–206 also detected an additional protein of ≈110 kDa. Additional western blot (Figures S1–S4) and immunofluorescence analyses (Figures S5 and S6) performed in PLK1‐silenced cells generated by RNA interference confirm the high specificity of the 3F8 and 13E8 clones for PLK1, whereas 35–206 still detects an additional species even in PLK1‐silenced cells.

**FIGURE 4:**
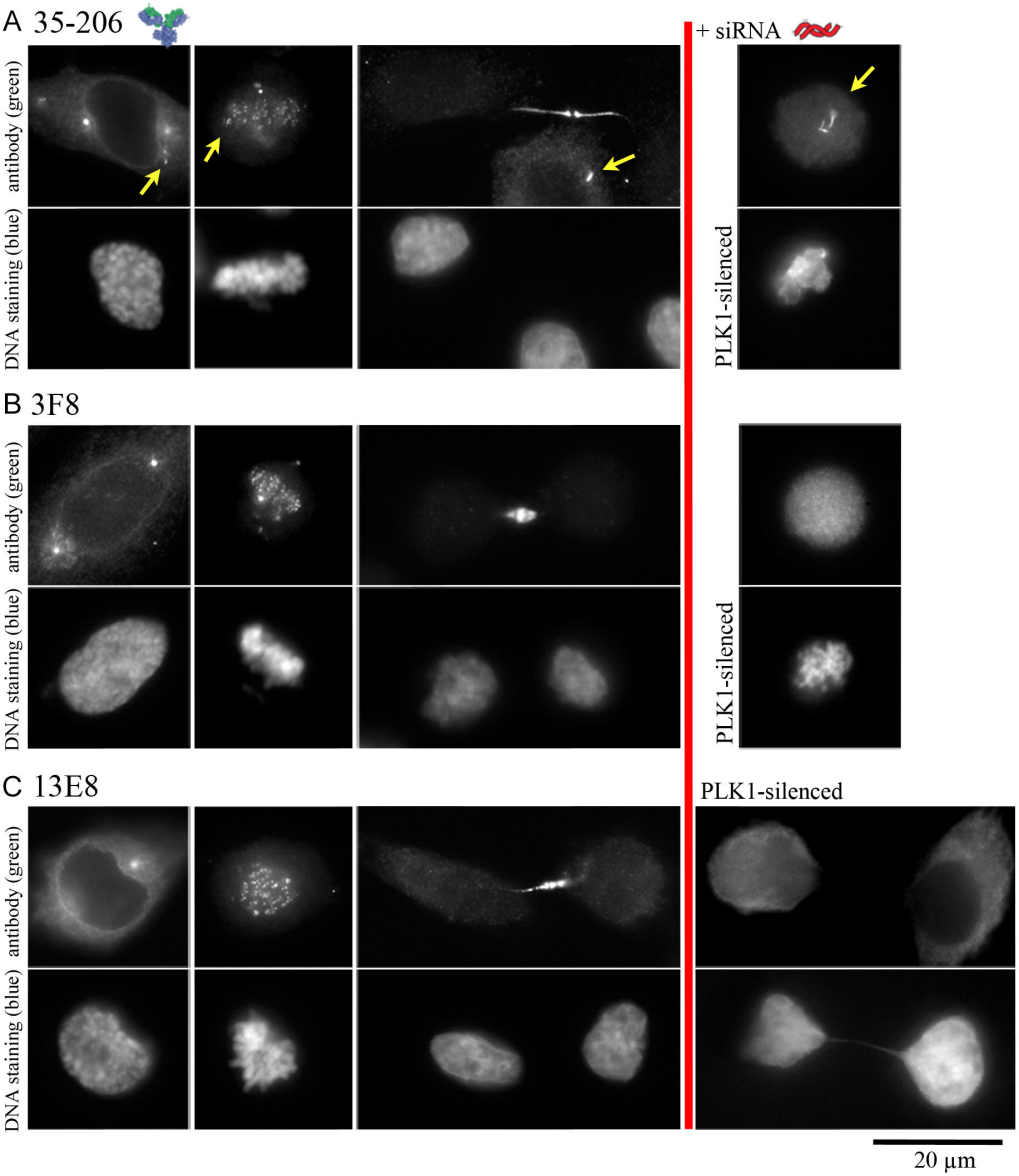
Immunofluorescence labeling of the indicated antibody following cytosolic delivery by electroporation. HeLa cells (1 × 10^5^) were electroporated in the presence of 1.5 µM mAb using three 10 ms pulses at 517 V·cm^−1^, then diluted in culture medium and incubated for 18 h. Cells were fixed with 4% PFA and permeabilized with 0.1% Triton X‐100. Nuclei were stained with Hoechst 33 242 (blue). The mAb was detected with an Alexa Fluor 488‐conjugated anti‐mouse secondary antibody (green). Yellow arrows indicate fluorescent labeling apparently unrelated to PLK1. PLK1 expression was silenced using PLK1‐targeting siRNA co‐delivered with the mAb during electroporation.

For subsequent experiments involving living cells, in which electroporation was used as the intracellular delivery method, commercial antibodies were purified by gel filtration to remove the preservative sodium azide and then concentrated to 6.6 µM in PBS using centrifugal devices (Figure S7). HeLa cells were also synchronized in early S phase using a double thymidine block. Antibodies were delivered immediately after release from the DNA replication block by electroporation. Electroporation parameters were optimized to induce transient membrane permeabilization and effective antibody inflow into the cytosol while allowing rapid cell recovery.^33^ Cells were grown for an additional 18 h, fixed, and the intracellular distribution of anti‐PLK1 was detected using an Alexa Fluor 488–conjugated secondary antibody and fluorescence microscopy. Images representing different stages of the cell cycle were selected (Figure 4).

Overall, the three antibodies displayed a similar intracellular labeling pattern. This pattern was highly consistent with the known subcellular localization of PLK1 during progression into mitosis [2, 5, 15, 21]. Centrioles were labeled during prophase, centromeres during late prophase or metaphase, and midbodies during cytokinesis. Depleting intracellular PLK1 by RNA interference to less than 10% resulted in a marked reduction of the labeling pattern. Consistent with the previously observed recognition of an additional protein by 35–206, this antibody also labeled fiber‐like assemblies (yellow arrows, Figure 4A) that remained detectable even in PLK1‐silenced cells. A similar labeling pattern was observed following electroporation of U2OS cells (Figure S8). Additional images, illustrating the distribution of the cytosolic monoclonal antibodies (mAbs) in HeLa cells during cytokinesis, are shown in Figure S9. The application of electric pulses, enabling the cytosolic entry of antibodies, was required to observe PLK1 labeling (Figures S8 and S9).

**FIGURE 5:**
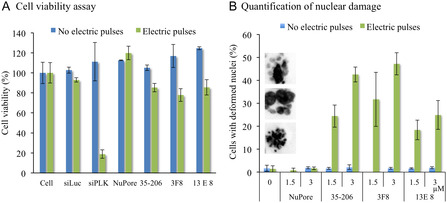
Impact of cytosolically delivered mAbs on HeLa cell viability. (A) Cell viability assessed by MTT assay 36 h after electroporation (3 µM mAb, three 10 ms pulses at 517 V cm^−1^). Results are presented as mean ± SD (*n* = 3). (B) Analysis of nuclear damage. HeLa cells were electroporated with the indicated mAbs (1.5 or 3 µM) and cultured for 48 h. Cells were fixed with 4% PFA, and nuclei were stained with Hoechst 33242 for morphology analysis. Results are presented as mean ± SD (*n* = 2).

Having established that the 3F8 mAb binds PLK1 upon delivery into living cells, we next assessed the effects of electroporation and selective targeting on cell viability. Flow cytometry quantification of cell‐surface Annexin V staining and propidium iodide uptake showed that approximately 80% of cells remained viable 24 h post‐treatment in the control groups (untreated cells; electroporation with control small interfering RNA (siRNA) or antinuclear pore (NuPore) mAb) as well as in the PLK1‐targeting groups (40 nM Volasertib; electroporation with PLK1‐targeting siRNA or 3F8) (Figure S12). These results indicate that neither the electroporation procedure nor PLK1‐targeting treatments induced substantial apoptosis 24 h post‐treatment. Although apoptosis was not detectable 24 h post‐treatment, a reduction in mitochondrial membrane potential was observed in cells treated with Volasertib, PLK1‐targeting siRNA, or 3F8, indicating the early onset of new processes impairing cell viability (Figure S13). At 36 h post‐electroporation, an overall cell viability assay was performed using the MTT test. An approximately 80% reduction in cell viability was observed upon silencing PLK1 expression with PLK1 siRNA. A more modest, approximately 20% decrease in cell viability was induced by PLK1‐targeting mAbs, whereas control conditions remained unaffected (Figure 5A). Overall, these data indicate that the cells recovered from the electric shock and transient membrane permeabilization. Moreover, cell function began to be impaired 24 h post‐treatment and over time, consistent with the mechanism of PLK1‐targeting compounds, which act during mitosis and induce cell death after a delayed process.

The selectivity of the PLK1‐targeting compounds for the cell division process was then assessed by observing nuclear morphology. HeLa cells were electroporated with anti‐NuPore or anti‐PLK1 mAbs (1.5 or 3 µM), cultured for 48 h, and then fixed and stained with Hoechst 33 242. Nuclear morphology analysis (Figures 5B and S12) revealed that most control nuclei retained the rounded shape typical of interphase, whereas 3F8 and 35–206 induced some aberrant nuclear morphologies, as illustrated in the images in Figure 5B, that are similar to those observed in PLK1‐depleted or PLK1‐inhibited cells [37]. The proportion of abnormal nuclei increased from ≈30% to ≈50% as the mAb dose was doubled, while 13E8 was slightly less effective (≈25%–30% abnormal nuclei).

**FIGURE 6:**
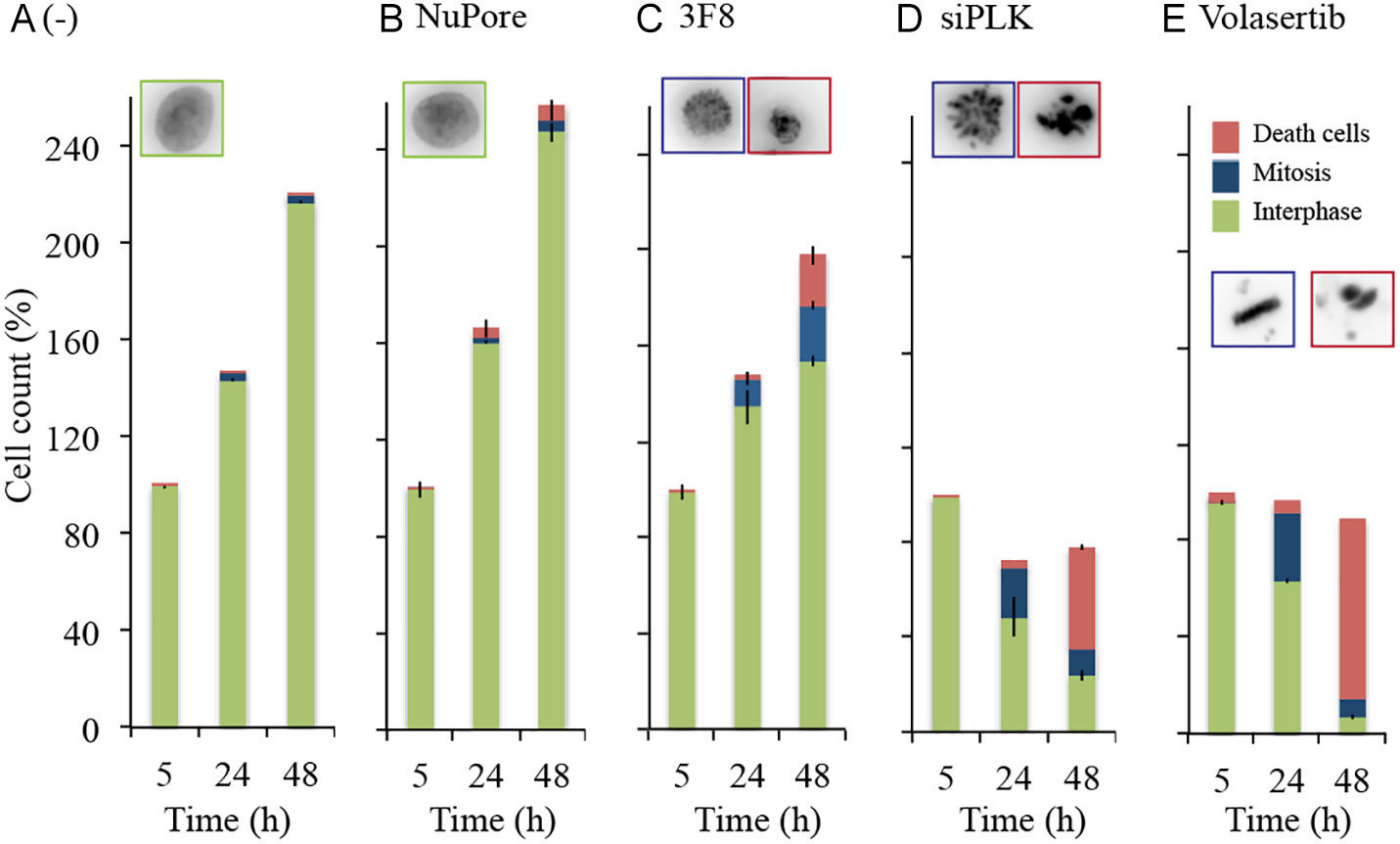
Time‐lapse imaging of nuclear morphology in H2B‐GFP HeLa cells following electroporation. Cells were electroporated without antibody (A), with 3 µM antinuclear pore antibody (B), 3 µM 3F8 (C), or 2 µM PLK1 siRNA (D). Volasertib (40 nM) was added to nonelectroporated cells as a control. At the indicated time points, nuclei from the same movie were counted and classified (see insets and outlines) as interphasic (green), mitotic (blue), or fragmented ‐ dead cells (red). Results are presented as mean ± SD (*n* = 2 images, except for the volasertib condition, *n* = 1).

To visualize more precisely the impact of anti‐PLK1 compounds on cell division and nuclear morphology, H2B‐GFP HeLa cells [38, 39] constitutively expressing a histone H2B‐GFP fusion protein were electroporated with PLK1‐targeting macromolecules (siRNA, mAb 3F8) and control ones (Luciferase siRNA, anti‐NuPore mAb). Incubation with 40 nM Volasertib was included as an additional condition to compare PLK1‐targeting strategies. As described above, cells were synchronized in early S phase and subjected to electric pulses immediately after release from the replication block. Under these conditions, most cells entered mitosis 11 h post‐release. Time‐lapse fluorescence imaging of nuclei in living cells was initiated 5 h post‐treatment. The resulting image sequences were analyzed 5, 24, and 48 h after electroporation to assess cell proliferation and cell cycle status based on cell counts and nuclear morphology, with nuclei classified as interphasic, mitotic, or fragmented (e.g., dead cells) (Figures 6, S15, and S16). In untreated cells or in cells electroporated with the anti‐NuPore antibody, cell counts increased at a similar rate. Most cells completed the first cell division by 24 h, and a fraction proceeded into a second division by 48 h, resulting in more than a doubling of the total cell number. In both groups, fewer than 10% of cells were in mitosis or displayed fragmented nuclei at either time point. In contrast, cells electroporated with PLK1 siRNA or treated with Volasertib behaved markedly differently: the first cell division was fully arrested at 24 h, with no apparent increase in cell counts, and approximately 20% of cells were arrested in mitosis. By 48 h, most cells exhibited fragmented nuclei, representing 54% and 83% of the population in PLK1‐silenced and Volasertib‐treated cells, respectively. Cells treated with 3F8 displayed an intermediate phenotype. At 24 h, the number of cells increased, but by approximately 30% less than in the control conditions. About 10% of the cells were in mitosis. At 48 h, the proportions of mitotic cells (23%) and dead cells (20%) were higher, although they did not reach the levels observed with siRNA‐mediated PLK1 silencing or treatment with the PLK1 kinase inhibitor Volasertib. We next quantified the duration of the mitosis from the movies (Figure S17). Nontreated cells, cells electroporated with Luciferase siRNA, and cells containing anti‐NuPore mAbs completed mitosis in ≈50 min, whereas anti‐PLK1 3F8‐containing cells exhibited a prolonged ANOVA statistical analysis indicated that these differences were highly significant (*q*‐value of 10^−16^ comparing the anti‐PLK1 condition to the anti‐NuPore one). The prolonged mitosis was followed either by : 1. ‐ successful cell division with continuation of a normal cell cycle; 2. ‐ apoptosis, as observed in siRNA‐mediated PLK1‐silenced cells; and 3.‐ cell division immediately followed by fragmentation of the daughter nuclei (Figures S18 and S19).

**FIGURE 7:**
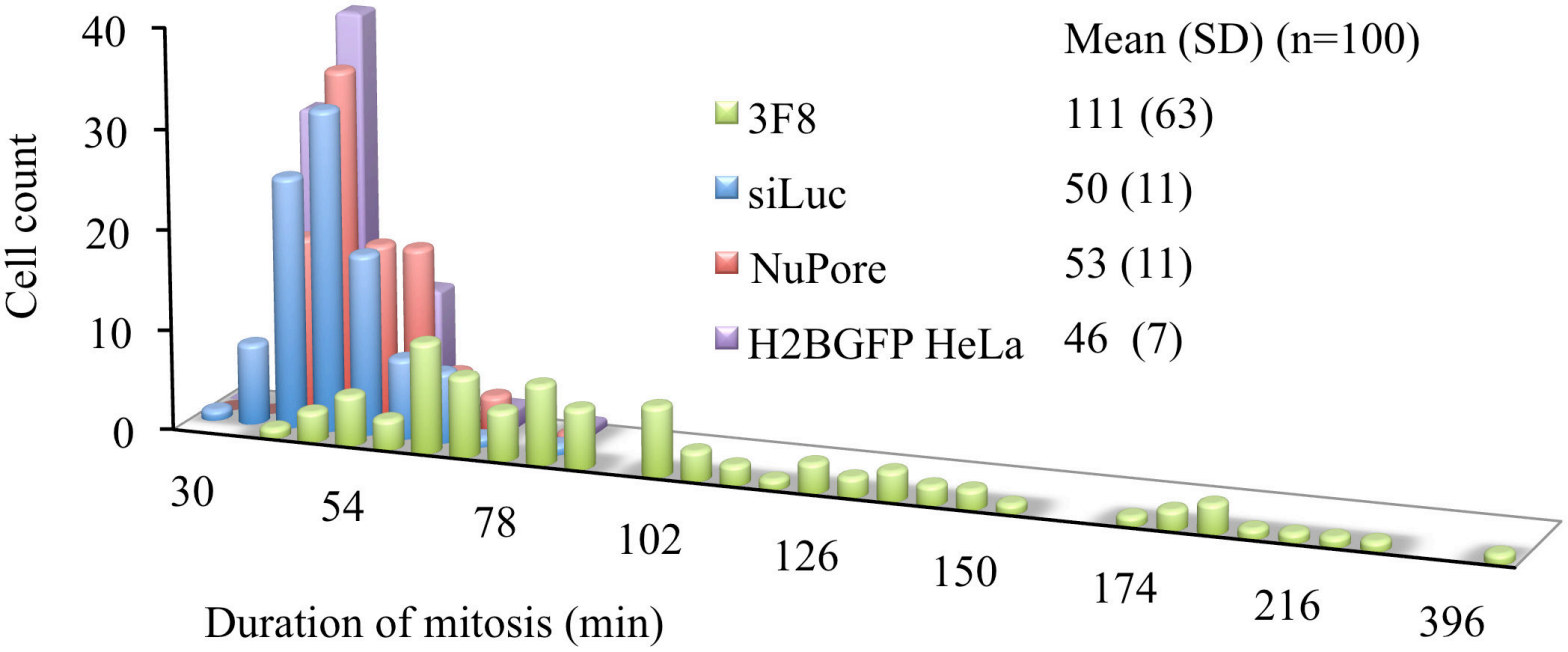
Distribution of mitotic duration across individual cells (*n* = 100) measured by time‐lapse microscopy. Synchronized H2BGFP‐HeLa cells were electroporated with siLuc siRNA (2 µM), antinuclear pore (3 µM), or 3F8 antibodies (3 µM).

**FIGURE 8:**
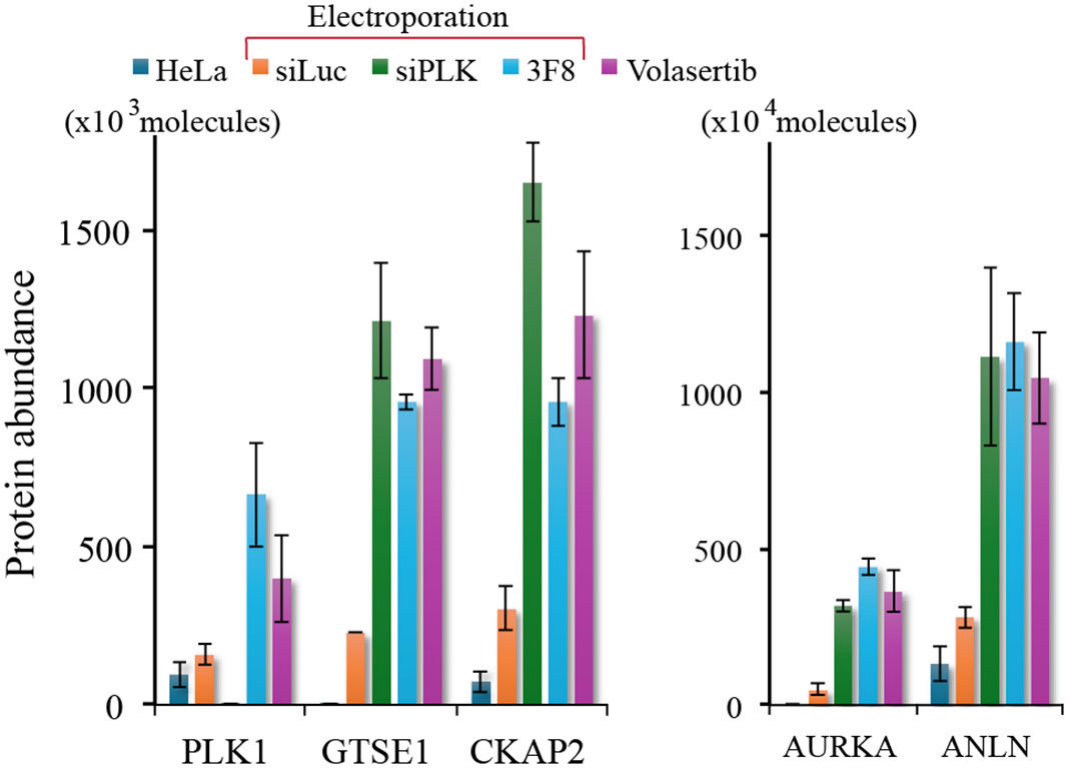
Abundance of PLK1, GTSE1, CKAP2, AURKA, and ANLN in HeLa cells 24 h after treatment. Synchronized HeLa cells were treated with volasertib (40 nM) or electroporated with luciferase siRNA (2 µM), PLK1 siRNA (2 µM), or anti‐PLK1 mAb 3F8 (3 µM). Cells were harvested for proteomic analysis 24 h post‐electroporation. Data are mean ± SD (*n* = 3).

The outcome of PLK1 targeting by siRNA, antibody, or kinase inhibition on the cell activity was next indirectly evaluated by proteomic analyses 24 h post‐treatment. The 3F8 antibody (3 µM) was selected as the representative anti‐PLK1 mAb because 13E8 is contaminated with BSA, and 35‐206 is less specific. Antibodies and siRNA require cytosolic delivery, currently accomplished via electroporation. The effect of electroporation was monitored with luciferase‐targeting siRNA. Luciferase siRNA was preferred over the anti‐NuPore antibody in this pilot experiment because it has no endogenous target. Analysis of variance (ANOVA) with Benjamini–Hochberg correction for multiple testing (false discovery rate, FDR = 0.01) on the proteomic data identified 49 proteins with significant variance. Euclidean hierarchical clustering (Figure S20) revealed that electroporation is not completely innocuous but shares a proteomic profile very close to that of untreated cells. In contrast, a more pronounced difference from controls was observed for a cluster of 22 proteins up‐regulated in samples treated with PLK1‐targeting agents. Functional analysis using STRING (string‐db.org) indicated that 20 of these proteins are involved in spindle elongation and PLK‐mediated processes, with the remaining two proteins, KNSTRN and PCOLCE, not associated with these pathways. Pairwise ANOVA comparisons with either untreated or siLuc‐electroporated HeLa cells as references revealed that GTSE1, AURKA, CKAP2, and ANLN were significantly up‐regulated upon PLK1 targeting (*q*‐values < 10^−4^). The quantification of PLK1 and selected proteins is shown in Figure 8. The proteomic data estimate endogenous PLK1 abundance to range from 80 000 to 600 000 molecules per cell, consistent with a previous report (≈150 000 PLK1 molecules per cell [40]) and with expected cell‐cycle variation. ANLN and CKAP2 are known to play important roles in cytokinesis. In addition, strong evidence indicates that AURKA, GTSE1, and PLK1 are part of the same complex. Together, these data further support a specific involvement of intracellular 3F8 in PLK1‐mediated mitotic processes.

**FIGURE 9:**
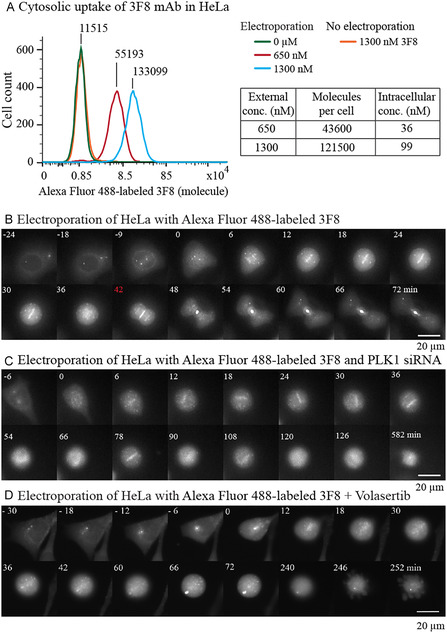
Quantification of 3F8 internalization in HeLa cells (A) and time‐lapse imaging of 3F8 binding to PLK1 in living cells (B–D). 3F8 was labeled with Alexa Fluor 488 and electroporated into synchronized HeLa cells. (A) Flow cytometry analysis performed 5 h after electroporation. Values above the curve indicate median fluorescence (*n* = 10 000 cells). The intracellular concentration of 3F8 was estimated assuming a cell volume of 2 pL. (B–D) Time‐lapse montage of a HeLa cell undergoing mitosis in the presence of Alexa Fluor 488–conjugated 3F8 (1.5 µM) (B), 3F8 (1.5 µM) co‐delivered with PLK1 siRNA (2 µM) (C), or 3F8 (1.5 µM) in the presence of Volasertib (40 nM) (D).

Clones 3F8 and 35‐206 were then fluorescently labeled on lysine side‐chain amines by reaction with Alexa Fluor 488N‐hydroxysuccinimidyl‐ester. The removal of unreacted dye was performed by gel filtration. The average numbers of Alexa Fluor 488 molecules conjugated to 3F8 and 35–206 were determined to be 2 and 4.28, respectively (Figure S21). These Alexa Fluor 488–labeled antibodies were used to quantify their entry into HeLa cells following electroporation (Figure 9A). Flow cytometry analysis, calibrated using fluorescent reference beads in the green channel, indicated that electroporation enabled the delivery of approximately 50 000 and 120 000 antibodies into the cytosol at external concentrations of 650 and 1300 nM, respectively. In contrast, incubation of the antibodies with cells in the absence of electric pulses did not alter cellular autofluorescence, indicating negligible surface binding. Due to technical limitations, Alexa Fluor 488 antibody conjugates could not be prepared at higher concentrations. Linear extrapolation suggests that an external concentration of 3 000 nM during transient membrane permeabilization by electroporation could, in principle, yield an inflow of ≈200 000 antibody molecules per cell. This delivery efficiency results in an intracellular amount of anti‐PLK1 antibody comparable to, although still slightly lower than, the average cellular abundance of PLK1. The emergence of mitotic perturbation under these near‐stoichiometric conditions supports a specific functional interplay between the two partners.

In a final experiment, intracellular targeting of PLK1 by the anti‐PLK1 antibodies was investigated in living HeLa cells using Alexa Fluor 488–labeled antibody 3F8, delivered alone or in combination with PLK1 siRNA or Volasertib (Figure 9B–D, Movies M3‐HeLa‐3F8*, M4‐HeLa‐SiPLK1‐3F8*, M15‐HeLa‐3F8*Volasertib). During prophase, 3F8 localized to the centrioles and the perinuclear region (Figure 9B). Upon nuclear envelope breakdown, the fluorescence was redistributed toward centromeres as mitosis progressed. The labeling remained in the cell center during anaphase, and the labeled elements concentrated into a midbody during daughter cell separation, with most of the fluorescence accumulating at the midbody. This ordered and specific dynamic localization mirrors that previously reported for YFP–PLK1 fusions [16, 17, 18, 41], demonstrating that cytosolic antibodies specifically bind endogenous PLK1 throughout the mitotic process.

PLK1 depletion by RNA interference (siRNA‐mediated silencing rarely exceeds ≈95%) resulted in cells entering mitosis and exhibiting weaker, specific labeling at the center of the mitotic spindle (Figure 9C). The cells subsequently underwent a prolonged metaphase‐like arrest that culminated in apoptosis. Co‐treatment with Volasertib revealed binding of 3F8 to intracellular PLK1 at centrioles during the G2/M transition, along with a slightly altered labeling pattern. A similar mitotic trajectory, in which prolonged metaphase arrest is followed by apoptosis, was observed (Figure 9D). The extent of apoptosis appeared higher under co‐treatment conditions, suggesting a possible complementary effect. Similar labeling patterns and effects on HeLa cell cycle progression were observed using the Alexa Fluor 488–labeled antibody 35–206 in co‐treatment experiments (Figures S22–S28). Intracellular targeting of PLK1 and real‐time labeling were also confirmed in U2OS cells (Figure S29).

## 3 Conclusion

Our study establishes a paradigm for intracellular targeting of PLK1 using monoclonal antibodies directed against its IDL. Intracellular delivery of antibodies and their specific binding to the PLK1 IDL in living cells enables visualization of PLK1 dynamics during mitosis. When the number of intracellular antibodies approaches that of endogenous PLK1, antibody binding to the IDL also appears to perturb its mitotic orchestration. This dual capacity for visualization and mild functional perturbation establishes these intracellular monoclonal antibodies as a powerful tool for probing the PLK1‐orchestrated protein–protein interaction network from within the cell. More broadly, our findings open the way to therapeutic strategies that move beyond classical small‐molecule inhibitors. Avenues for improvement remain. Engineering smaller antibody derivatives could increase delivery efficiency, thereby ensuring that they outnumber PLK1 molecules. Electroporation is not the only available option. Tailored delivery systems or new formulations optimized for engineered proteins could also enable in vivo delivery.

## 4 Experimental Section

### 4.1 General

Unless otherwise indicated, chemicals were purchased from Sigma–Aldrich. Plastic tubes were certified RNase‐free and sterilized by autoclaving. Antibodies were stored at 0°C–4°C. Water was deionized using a Millipore Milli‐Q apparatus. PBS was sterilized by autoclaving. The monoclonal anti‐PLK1 antibodies 13E8 (Ref. MA1‐848, batch UA273497) and 35–206 (Ref. 37‐7000, batch WD329582) were obtained from Invitrogen. Anti‐PLK1 3F8 (Ref. 627702, batch B290751) and antinuclear pore complex proteins (Ref. 902902, batch B264669) were purchased from BioLegend (www.biolegend.com,, San Diego, CA, USA). Prior to use, monoclonal antibodies were purified from sodium azide by gel filtration chromatography (PD‐10 desalting column, VWR, Ref. 17‐0851‐01) and concentrated in PBS using a 0.5 mL Microcon 100 kDa centrifugal filter device (Merck, Molsheim, Ref. UFC5100BK, 5 × 0.5 mL PBS). Antibodies were assessed by SDS‐PAGE and Coomassie Blue staining. Concentrations were determined by Bradford assay or UV spectrophotometry using an extinction coefficient at 280 nm (ε_280_) of 210 000 M^−1^ cm^−1^. A 100 mM thymidine stock solution was prepared in water and sterilized by filtration using a 0.22 µm filter. U2OS cells stably expressing EGFP‐luc were generated as previously described by transfection of U2OS (ATCC HTB‐96) with the pEGFPLuc plasmid using a modified PEI protocol, followed by selection with G418 [42, 43]. siRNAs were purchased from Eurogentec (Seraing, Belgium), annealed at 90 µM (1.23 µg µL^−1^) and stored at −80°C in aliquots. The sense (S) and antisense (AS) sequences of the siPLK duplex were: 5’‐AGAU CACC CUCC UUAA AUAU U (S) and 5’‐UAUU UAAG GAGG GUGA UCUU U (AS) [44]. The siLuc duplex sequences were: 5’‐GAUU AUGU CCGG UUAU GUAU U (S) and 5’‐UACA UAAC CGGA CAUA AUCU U (AS) [45]. Underlined nucleotides denote 2’OMe modifications. Stock solutions were prepared in RNase‐free water at 100 µM.

### 4.2 Cell Culture

Human cervical carcinoma cells HeLa (ATCC Cat# CCL‐2, RRID:CVCL_0030), histone‐green fluorescent protein expressing HeLa cells H2BGFP‐HeLa (SCC117, Merck Millipore, RRID:CVCL_ZM02), human osteosarcoma cells U2OS (ATCC HTB‐96) U2OS (RRID:CVCL_0042) and human osteosarcoma cells expressing both luciferase (LUC) and green fluorescent protein (EGFP) (EGFPLuc‐U2OS, produced in our laboratory) were cultured adherently on plastic substrates (75 cm^2^ Falcon tissue culture flasks) in high‐glucose Dulbecco’s Modified Eagle Medium (DMEM) supplemented with 10% fetal bovine serum (FBS) (Perbio, Brebières, France), 2 mM L‐glutamine, 100 U/mL penicillin G, and 100 μg/mL streptomycin.

Cells were detached at 80%–90% confluence using 0.05% trypsin. Passage number was maintained below 12. Cell counts were performed using Luna cell counting slides (Logos Biosystems) and the Logos counter.

### 4.3 Fluorescence Immunolabeling

Freshly trypsinized cells (5 × 10^4^ cells in 0.5 mL medium) were plated onto 13 mm diameter cover glasses (N°1, Ref. ECN 631‐1578) in 24‐well plates and allowed to adhere for at least 9 h. Cells were washed with PBS (2 × 0.8 mL) and fixed with 4% paraformaldehyde (PFA) in 0.1 M sodium phosphate buffer (pH 7.6) for 30 min. Fixed cells were washed (3 × 0.8 mL PBS), permeabilized with 0.1% Triton X‐100 in PBS for 5 min, and incubated with primary antibodies (0.02 µg mL^−1^ in PBS containing 10% FBS, 40 min), followed by secondary Alexa488‐conjugated anti‐mouse antibodies (0.02 µg mL^−1^ in PBS containing 10% FBS, 40 min). Nuclei were stained with Hoechst 33242 (1 µg mL^−1^ in PBS, 20 min). After washing (3 × 0.8 mL PBS, 5 min each), cover glasses were mounted on microscope slides (3 × 1 inch, knittelglass.com) using Fluoromount‐G (Southern Biotech, Ref. 0100‐01, batch I2819‐WC79B) [46]. Fluorescence imaging was performed using a Leica DM5500B microscope equipped with HCX PL Apo 63×/1.40 oil PH3CS and HCX PL Fluotar 40×/0.75 PH2 objectives and a Leica DFC350FX camera.

### 4.4 Synthesis of mRNA

DNA sequences encoding PLK1 fragments were amplified from the plasmid‐68132‐CeruleanPLK1 vector (addgene) with the following primers: T7‐PLK1‐IDL‐for AATTA ATACG ACTCA CTATA GGGA GAAAG GAGAT ATCCA TGctt aatga cgagt tcttt a and either PLK1‐IDL‐Rev T_18_‐agcct cctct tgcct gaccag ccc to amplify the IDL coding region or the PLK1‐Cterm‐Rev T_18_‐aacaa caaca attgc attca tttta tg. PCR fragments were purified and used for in vitro transcription as previously described [7, 47]. 5×10^5^ HeLa cells were transfected using 5 µg mRNA by electroporation and grown for 24 h in culture medium without antibiotics for Western blot analysis.

### 4.5 Western Blot Analysis

Culture media were removed and cells washed with PBS, then harvested with 0.05% trypsin (80 µL). Cells were collected by centrifugation (400 g, 5 min), resuspended in PBS (0.5 mL), and pelleted again (400 g, 5 min). Cell pellets were lysed in 50 mM Tris‐HCl (pH 8) containing 150 mM NaCl, 0.1% SDS, 1% NP‐40, 1 mM DTT, and protease inhibitors (Complete, Roche). Protein concentrations were determined by Bradford assay. Lysates (50 µg) were subjected to 12% SDS‐PAGE and transferred onto nitrocellulose membranes. PLK1 and EGFPLuc were detected using monoclonal anti‐PLK1 (3F8, BioLegend) and HRP‐conjugated anti‐GFP antibody (Miltenyibiotec Ref. 130‐091‐833), followed when required by HRP‐conjugated secondary antibodies (goat anti‐mouse IgG‐HRP, Ref. UP446330, batch K10L222; goat anti‐rabbit IgG‐HRP, Ref. UP511380, batch L11L206) using chemiluminescence (ECL, Millipore) or IRDye800‐conjugated anti‐mouse secondary antibodies (Licor Bio, Ref. 926‐32210). Membranes were scanned with the LI‐COR Odyssey infrared imaging system.

### 4.6 Cytosolic Delivery

Antibody delivery was performed using the Neon transfection system with 10 µL syringes [48]. Typically, 2×10^5^ cells in 10 µL PBS were mixed with 4 µL of antibody solution (5.3 or 10.6 µM). 10 µL containing 1.5 or 3 µM antibodies were loaded into a Neon syringe and electroporated using three 10 ms pulses at 517 V cm^−1^. Cells were immediately diluted into 24‐well plates containing 0.5 mL pre‐warmed medium and cover glasses, then allowed to adhere. For gene silencing, siRNA concentrations in the syringe were 2 µM.

### 4.7 Cell Synchronization

Cells were synchronized using a double thymidine block [11, 49]. Thymidine was added to the culture medium at 2 mM from a 100 mM stock solution, and cells were incubated for 16 h at 37°C. After washing three times with PBS, cells were released in normal growth medium for 8 h, then subjected to a second thymidine block (2 mM, 16 h).

### 4.8 Fluorescent Labeling of mAb

Alexa Fluor 488 succinimidyl ester (0.65 µL of 1 mM in anhydrous DMF, 0.65 nmol) was mixed with 20 µL of mAb (1 µg µL^−1^ in PBS, 0.13 nmol). The reaction proceeded at 4°C for 16 h, after which the mixture was purified by gel filtration (Bio‐Rad P100 resin, 2 mL bed volume) and concentrated using a 0.5 mL Microcon 100 kDa centrifugal filter (Merck, Ref. UFC5100BK). Protein concentrations were estimated by densitometry after PAGE and Bradford assay. Alexa Fluor 488 concentration was estimated spectrophotometrically using an extinction coefficient at 488 nm (ε_488_) of 73 000 M^−1^ cm^−1^.

### 4.9 Live Cell Imaging

Post‐electroporation, cells were plated in Gibco FluoroBrite medium containing 10% serum into 35 mm or 8‐well glass‐bottom dishes and allowed to adhere for ≈4 h. Medium was then replaced with fresh FluoroBrite medium with 10% serum and antibiotics for live‐cell imaging. Time‐lapse microscopy was performed on an Olympus IX83 inverted microscope equipped with a Pecon cellVivo incubation chamber maintained at 37°C and 5% CO_2_. Images were typically acquired every 3 (U2OS‐3F8*), 6 (HeLa‐3F8*, U2OS‐siPLK3F8*, HeLa‐siPLK3F8*), 9 (H2BGFP‐HeLa) or 10 min (H2BGFP‐HeLa; Movies M9, 10 and 11) over a period of 24–48 h using a Hamamatsu Orca‐Fusion CMOS camera (C14440‐20UP) in phase‐contrast and fluorescence modes. Fluorescence excitation at 488 nm was provided by an XC‐LED‐385‐XYLIS lamp system with a GFP filter set (U‐F39002). Image acquisition was carried out using either a 40× objective (LUCPLFLN40XPH2/0.60) or a 20× objective (LUCPLFLN20XPH1/0.45). For each experimental condition, at least nine independent fields were recorded. Image analysis was performed using Olympus cellSens software (v3.2) and ImageJ/Fiji.

### 4.10 Statistical Analysis

ANOVA analyses for the Figure 7 were performed using the XLMiner analysis tool pack in Microsoft Excel. The statistical analyses of the proteomic data were performed by the IGBMC service.

## Supporting Information

Additional supporting information can be found online in the Supporting Information section.

## Author Contributions

**Clément Steyer** and **Mariel Donzeau** analyzed the binding specificity of the monoclonal antibody toward PLK1. **Karie Shen**, **Manuela Chiper**, and **Jean‐Christophe Amé** performed the time‐lapse microscopy experiments. **Guy Zuber** conducted additional experimental work and wrote the manuscript. **Manuela Chiper** contributed to the improvement of the final text.

## Funding

Agence Nationale de la Recherche (ANR‐10‐IDEX‐0002; ANR‐SFRI‐0012).

## Conflicts of Interest

The authors declare no conflicts of interest.

## Supporting information

Supplementary Material

## Data Availability

The data that support the findings of this study are available in the supplementary material of this article.
