# Supplementary material for: Monoclonal Antibodies Accessing the Cytosol of Living Cells and Binding to Polo‐Like Kinase 1 Interdomain Linker Affect Mitotic Behavior
Source: Chembiochem. 2026 Apr 29;27(9):e202500858. doi: 10.1002/cbic.202500858 (PMC13127698; doi:10.1002/cbic.202500858)
Supplement: Supplementary file 1 — Supplementary Material [file CBIC-27-e202500858-s001.pdf]

Supporting information

Monoclonal Antibodies Accessing the Cytosol of Living Cells and Binding to PLK1 Interdomain Linker Affect Mitotic Behavior

Clément Steyer, Jean-Christophe Amé, Mariel Donzeau, Karie Shen, Manuela Chiper, Guy Zuber

1. CNRS-Université de Strasbourg UMR 7242, Biotechnology and Cell Signaling, 300 Bld. Sébastien Brant BP 10413 F-67412 Illkirch

Email : zuber@unistra.fr

The supporting information contains 29 figures (Figures S1 to S29) and 2 tables.

Movies (AVI format) can be downloaded from the links

M1-U2OS-3F8* : https://seafile.unistra.fr/f/268b1484eebf45359f94/?dl=1

M2-U2OS-siPLK3F8* : https://seafile.unistra.fr/f/8b724e0ab6884076a07b/?dl=1

M3-HeLa-3F8* : https://seafile.unistra.fr/f/98cd54f0d717454b869c/?dl=1

M4-HeLa-siPLK3F8* : https://seafile.unistra.fr/f/347ebb2852624285b04b/?dl=1

M5-H2BGFPHeLa-siLuc : https://seafile.unistra.fr/f/ecdbb36613d348c58b8a/?dl=1

M6-H2BHeLa-3F8 : https://seafile.unistra.fr/f/ef65fc3fa3324dfeb7e0/?dl=1

M7-H2BGFPHeLa-NuPor : https://seafile.unistra.fr/f/a3a2f4bd69c948879fa4/?dl=1

M8-U2OS-35206* : https://seafile.unistra.fr/f/5fae2917be0946b1ab0a/?dl=1

M9-H2BGFPHeLa-PBS : https://seafile.unistra.fr/f/dd8ef7d0a9bf436bbc2b/?dl=1

M10-H2BGFPHeLa-3F8 : https://seafile.unistra.fr/f/f8fabc6aaf444f6593b8/?dl=1

M11-H2BGFPHeLa-NUP : https://seafile.unistra.fr/f/84c0464a129f4cbba36c/?dl=1

M12-H2BGFPHeLa-13E8after19h : https://seafile.unistra.fr/f/88cb3baf819743928316/?dl=1

M13-HeLa-35206* : https://seafile.unistra.fr/f/66741f5e3be24a5c9246/?dl=1

M14-HeLa-35206*Volasertib: https://seafile.unistra.fr/f/b951926b3e494770b621/?dl=1

M15-HeLa-3F8*Volasertib: https://seafile.unistra.fr/f/b951926b3e494770b621/?dl=1

Supplementary methods

Immunodetection analysis of the anti-PLK1 monoclonal antibodies. HeLa cells were synchronized in early S phase using a double thymidine block. Cells were trypsinized and electroporated for cytosolic delivery of siRNA or monoclonal antibodies (517 $V.cm^{-1}$; 3 pulses of 10 ms; 4 × 10^5 cells per condition; 3 µM mAb, 2 µM siRNA). After electroporation, cells were cultured in complete medium supplemented with 10% fetal bovine serum for 24 h. BI6727 (Volasertib; 20 mM stock solution in DMSO) was added 4 h after release from the thymidine block. Culture medium containing non/weakly adherent mitotic cells and adherent cells, detached with 0.05% trypsin (80 µL), were collected. Cells were pelleted by centrifugation (200 × *g*, 5 min) and washed five times with PBS (1 mL) by repeating the centrifugation step. Cell pellets were lysed in 100 µL of buffer containing 50 mM Tris-HCl (pH 8.0), 2% SDS, 10% glycerol, 4 mM DTT, and 0.01% (w/v) phenol red. Protein concentration was determined using the Bradford assay. Equal amounts of protein (20 µg) were resolved by 10% SDS-PAGE and transferred onto PVDF membranes (Amersham Hybond P, 0.45 µm) or nitrocellulose. Immunoblotting was performed using primary antibodies against PLK1 and GAPDH, followed by HRP-conjugated secondary antibodies and chemiluminescent detection. The ß-actin was detected with an HRP-conjugated primary antibody. Membranes were blocked with either 5% bovine serum albumin (BSA) or non-fat dry milk in PBS, and antibodies were diluted in 5% BSA. Washes were carried out in buffer containing 50 mM Tris-HCl (pH 7.6), 300 mM NaCl, and 0.1% Tween-20. The same lysates were used in all western blot analysis. The same protocol was used for western blots of U2OS cell lysates.

Evaluation of dead cells and apoptosis by flow cytometry. HeLa cells were synchronized in early S phase using a double thymidine block. Cells were trypsinized and electroporated for cytosolic delivery of siRNA or monoclonal antibodies (517 $V.cm^{-1}$; three pulses of 10 ms; 4 × 10^5 cells per condition; 3 µM mAb; 2 µM siRNA). After electroporation, cells were cultured in complete medium supplemented with 10% fetal bovine serum for 24 h. Volasertib (BI6727, Sigma Aldrich SML 3641,CAS number: 755038; 20 mM stock solution in DMSO) and Bleomycin A5 (EMD Millipore 203408, CAS Number 55658-47-4) (200 mM stock solution in PBS) were added 4 h after release from the thymidine block. Culture medium containing non- or weakly adherent mitotic cells was collected and combined with adherent cells detached using 0.05% trypsin (80 µL). Cells were then processed according to the manufacturer's protocol for the FITC Annexin V apoptosis detection kit (kit no. 556547, BD Pharmingen) and JC-10 (catalog no. 22204, AAT Bioquest). Flow cytometry analysis was performed at the IGBMC platform (Muriel Philipps) using the BD LSR Fortessa 20X.

Preparation of specimens for proteomic analysis. HeLa cells were synchronized in early S phase using a double thymidine block. Cells were trypsinized and electroporated for cytosolic delivery of siRNA or monoclonal antibodies (517 $V.cm^{-1}$; three 10 ms pulses; 8 × 10^5 cells per condition; 3 µM mAb; 2 µM siRNA). After electroporation, cells were cultured for 24 h in 24-well plates containing complete medium supplemented with 10% fetal bovine serum. Each experiment was performed in triplicate. Both detached and adherent cells were collected by trypsinization followed by centrifugation (200 × g, 5 min). Cell pellets were washed five times with PBS (1 mL). Pellets were then lysed in RIPA buffer containing a protease inhibitor cocktail (200 µL). Protein concentration was determined using a Bradford assay. Samples were subsequently submitted to the proteomics service of the IGBMC platform. There, the samples (15 µg each) were precipitated with trichloroacetic acid (TCA) and digested with trypsin. All samples (S01–S15) were injected on the LC–MS system in an incremental order

of three. A total of 3589 proteins were identified across all samples. Filtering based on the minimum number of valid values reduced the dataset to 3363 proteins.

Data Analysis. Log_2 transformation and median normalization were applied. The distribution of protein intensities across samples approximated a Gaussian distribution, with missing values representing only a minor fraction of the dataset. Sample S01 contained the highest proportion of missing values. Principal component analysis (PCA) performed on the 3363 quantified proteins revealed a broad dispersion of samples, indicating substantial variability among replicates. Differential protein abundance was assessed using analysis of variance (ANOVA) with Benjamini–Hochberg correction for multiple testing (false discovery rate, FDR = 0.01), which identified 49 significantly regulated proteins. Euclidean hierarchical clustering was subsequently performed on these 49 proteins using the ClustVis web tool (Metsalu and Vilo, *Nucleic Acids Res.*, 2015, 43, W566–W570; https://biit.cs.ut.ee). Pairwise ANOVA comparisons using either untreated HeLa cells or siLuc-electroporated HeLa cells as reference groups identified significant upregulation of **GTSE1, AURKA, CKAP2, and ANLN**, upon PLK1 targeting (q-values $< 10^{-4}$).

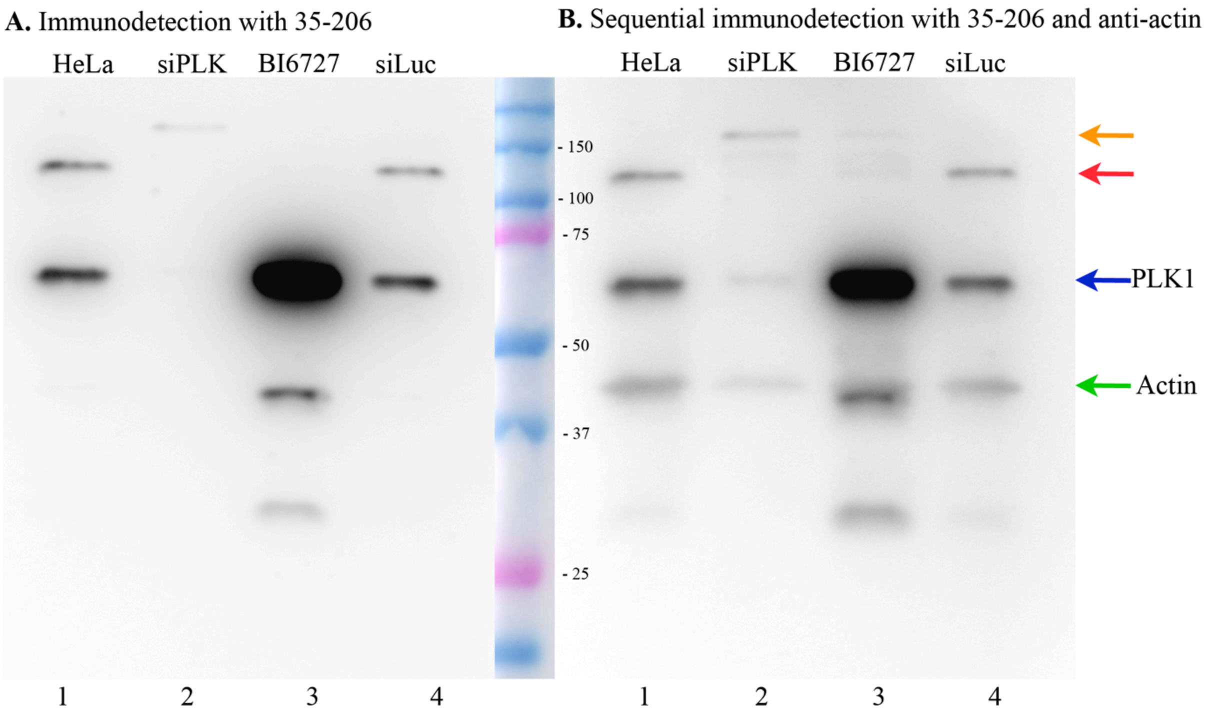

Figure S1. Immunodetection analysis of the anti-PLK1 monoclonal antibody 35-206 in HeLa cell lysates. (**A**) Western blot analysis using the monoclonal antibody 35-206. Lane 1: HeLa cells without electroporation; lane 2: HeLa cells electroporated with PLK1 siRNA (siPLK); lane 3: HeLa cells treated with 40 nM BI6727 (Volasertib) ; lane 4: HeLa cells electroporated with luciferase siRNA (control, siLuc). (**B**) The same membrane was reprobed with a HRP-conjugated anti-β-actin antibody as a loading control. Twenty micrograms of total cell lysate were loaded per lane. Red and orange arrows point to detection of unexpected bands. Important to note: a protein ladder (middle lane, "lane 0") was also loaded onto the gel and transferred onto the membrane. It was imaged using a standard camera, whereas anti-PLK1 antibody and actin were detected by chemiluminescence in a separate black-and-white image file. Automated reconstruction by the software did not yield high-quality results. To nonetheless provide an indication of the apparent molecular weights of the chemiluminescent bands, the ladder image was manually aligned alongside images A and B.

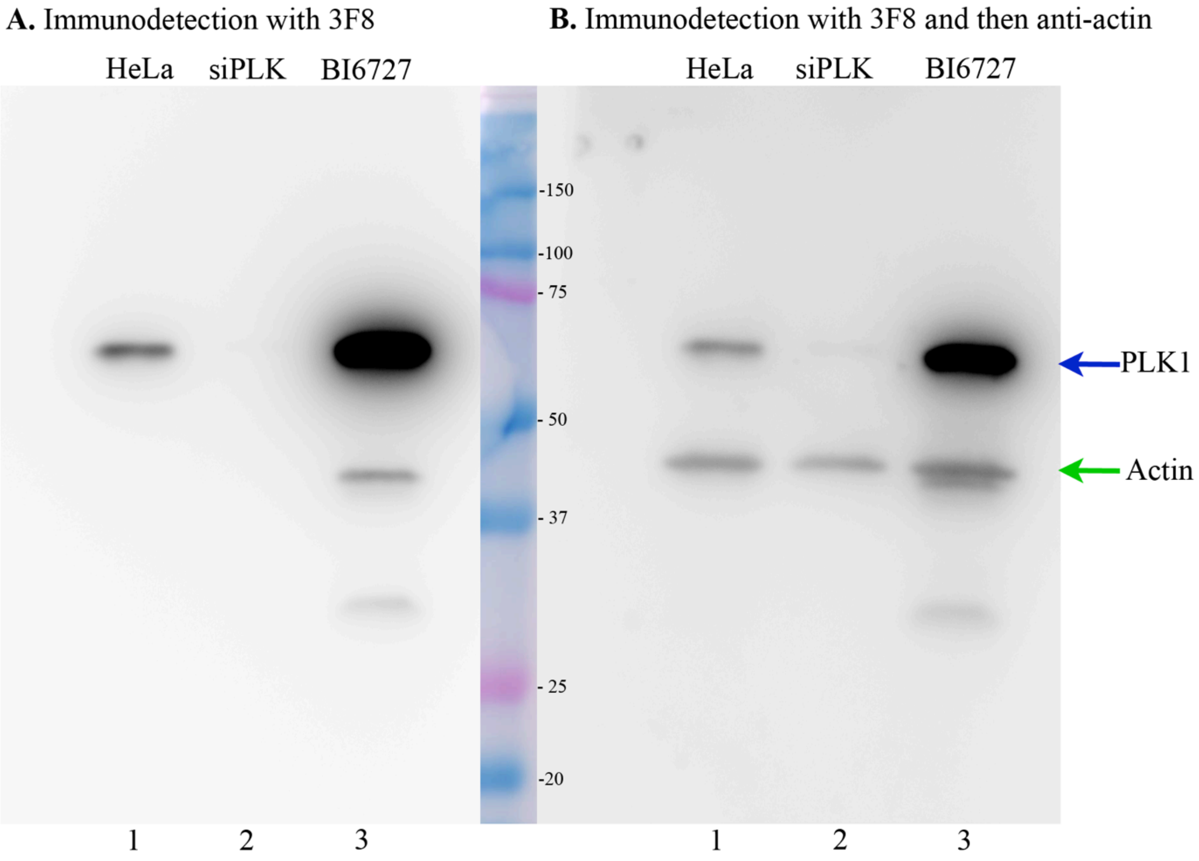

Figure S2. Immunodetection analysis of the anti-PLK1 monoclonal antibody 3F8 in HeLa cell lysates. (**A**) Western blot analysis using 3F8. Lane 1: HeLa cells without electroporation; lane 2: HeLa cells electroporated with PLK1 siRNA (siPLK); lane 3: HeLa cells treated with 40 nM BI6727 (Volasertib). (**B**) The same membrane was reprobed with a HRP-conjugated anti-β-actin antibody as a loading control. Twenty micrograms of total cell lysate were loaded per lane. Important to note: a protein ladder (middle lane, "lane 0") was also loaded onto the gel and transferred onto the membrane. It was imaged using a standard camera, whereas anti-PLK1 antibody and actin were detected by chemiluminescence in a separate black-and-white image file. Automated reconstruction by the software did not yield high-quality results. To nonetheless provide an indication of the apparent molecular weights of the chemiluminescent bands, the ladder image was manually aligned alongside images A and B.

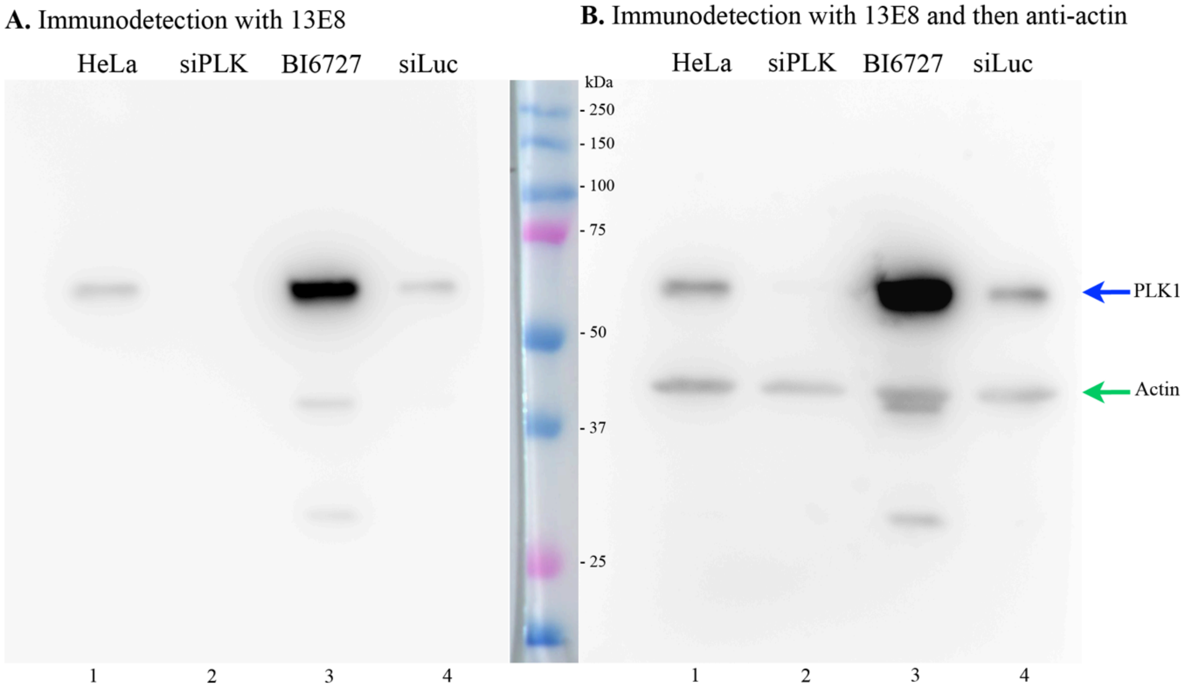

Figure S3. Immunodetection analysis of the anti-PLK1 monoclonal antibody 13E8 in HeLa cell lysates. (**A**) Western blot analysis using the monoclonal antibody 13E8. Lane 1: HeLa cells without electroporation; lane 2: HeLa cells electroporated with PLK1 siRNA (siPLK); lane 3: HeLa cells treated with 40 nM BI6727 (Volasertib); lane 4: HeLa cells electroporated with luciferase siRNA (control, siLuc). (**B**) The same membrane was reprobed with an HRP-conjugated anti-β-actin antibody as a loading control. Twenty micrograms of total cell lysate were loaded per lane. Blue and green arrows point to detection of expected PLK1 and actin bands, respectively. Important to note: a protein ladder (middle lane, "lane 0") was also loaded onto the gel and transferred onto the membrane. It was imaged using a standard camera, whereas anti-PLK1 antibody and actin were detected by chemiluminescence in a separate black-and-white image file. Automated reconstruction by the software did not yield high-quality results. To nonetheless provide an indication of the apparent molecular weights of the chemiluminescent bands, the ladder image was manually aligned alongside images A and B.

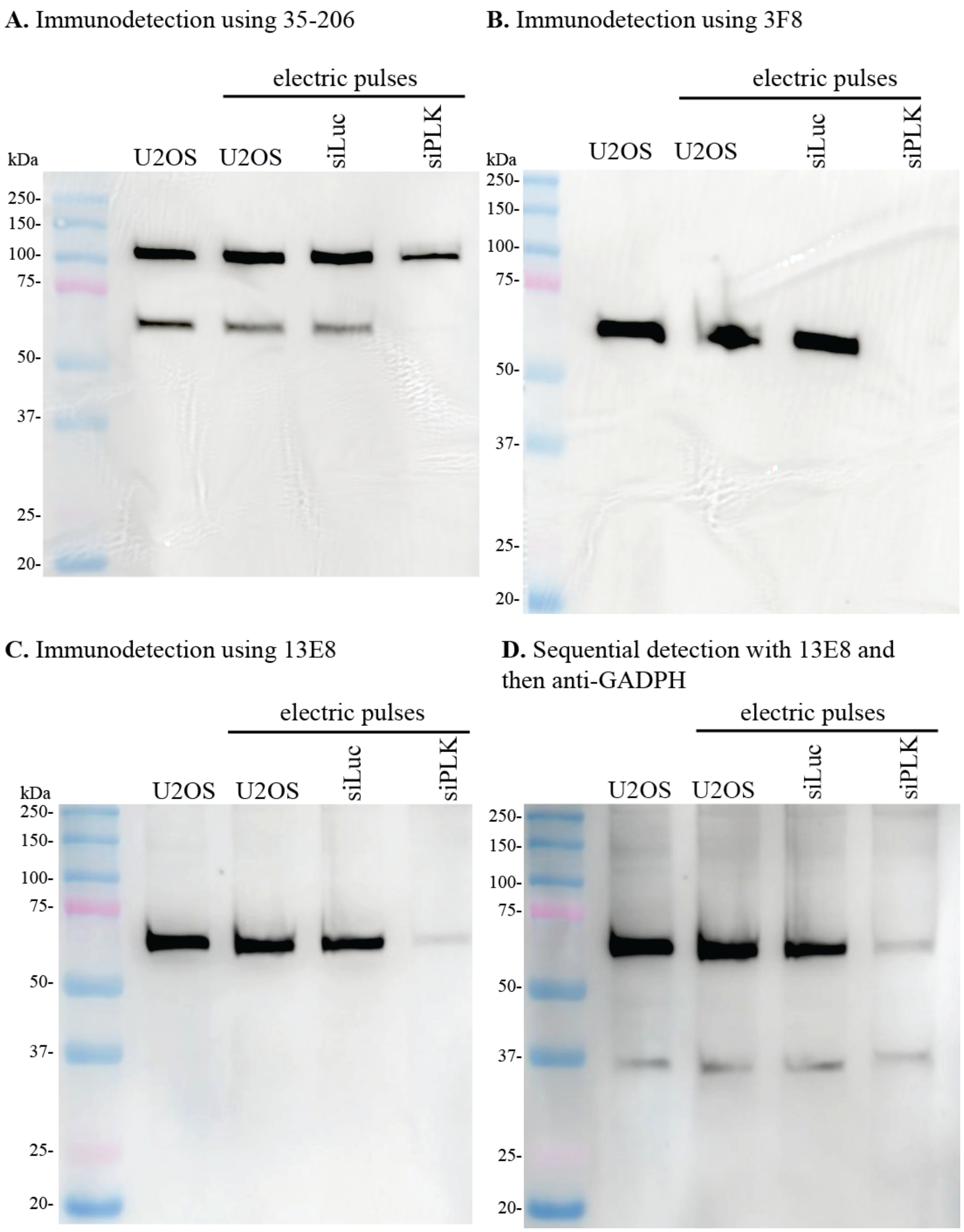

Figure S4. Immunodetection analysis of the anti-PLK1 monoclonal antibodies in U2OS cell lysates. Western blot analysis using the monoclonal antibody 35-206 (**A**), 3F8 (**B**) and 13E8 (**C**). Lane 1, Biorad protein ladder ; lane 2: U2OS cells without electroporation; lane 3: U2OS cells electroporated ; lane 4 : U2OS electroporated with luciferase siRNA (control, siLuc), lane 4 : U2OS electroporated with PLK1 siRNA (siPLK); (**D**) The same membrane (**C**) was reprobed with an anti-GADPH antibody and then with a secondary HRP-conjugated antibody as a loading control. Twenty micrograms of total cell lysate were loaded per lane. The same lysates were analyzed.

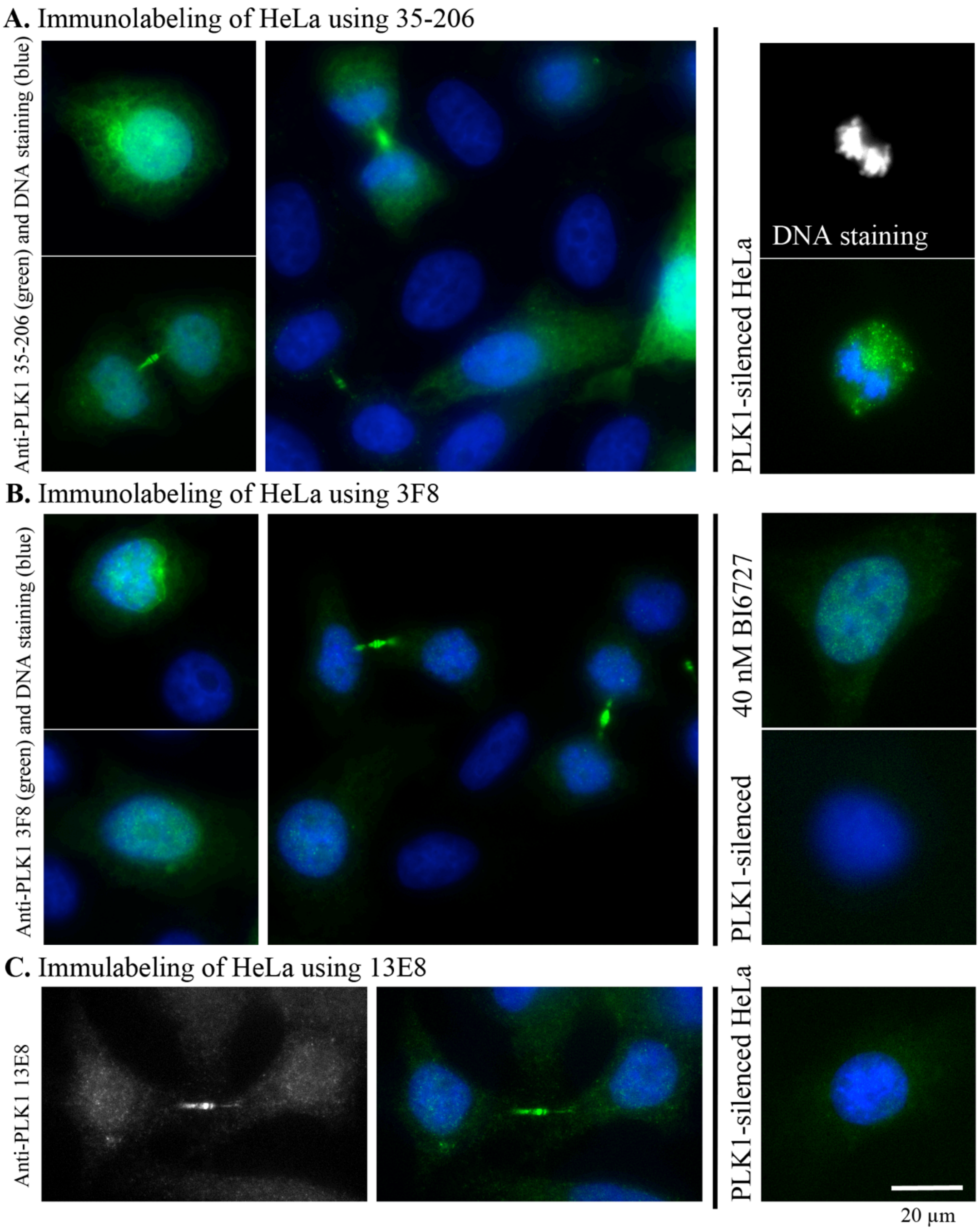

Figure S5. Classical immunofluorescence detection of PLK1 in HeLa cells. Wild type HeLa, PLK1-silenced HeLa (24 h post electroporation) or treated with 40 nM Volasertib (BI6727) were fixed with 4% (w/v) paraformaldehyde, permeabilized with 0.1% (w/v) Triton X-100, and incubated with the indicated primary anti-PLK1 antibody, followed by an Alexa Fluor 488–conjugated anti-mouse secondary antibody. Nuclei were stained with Hoechst 33242. Signals corresponding to PLK1 and DNA are shown in the green and blue channels, respectively.

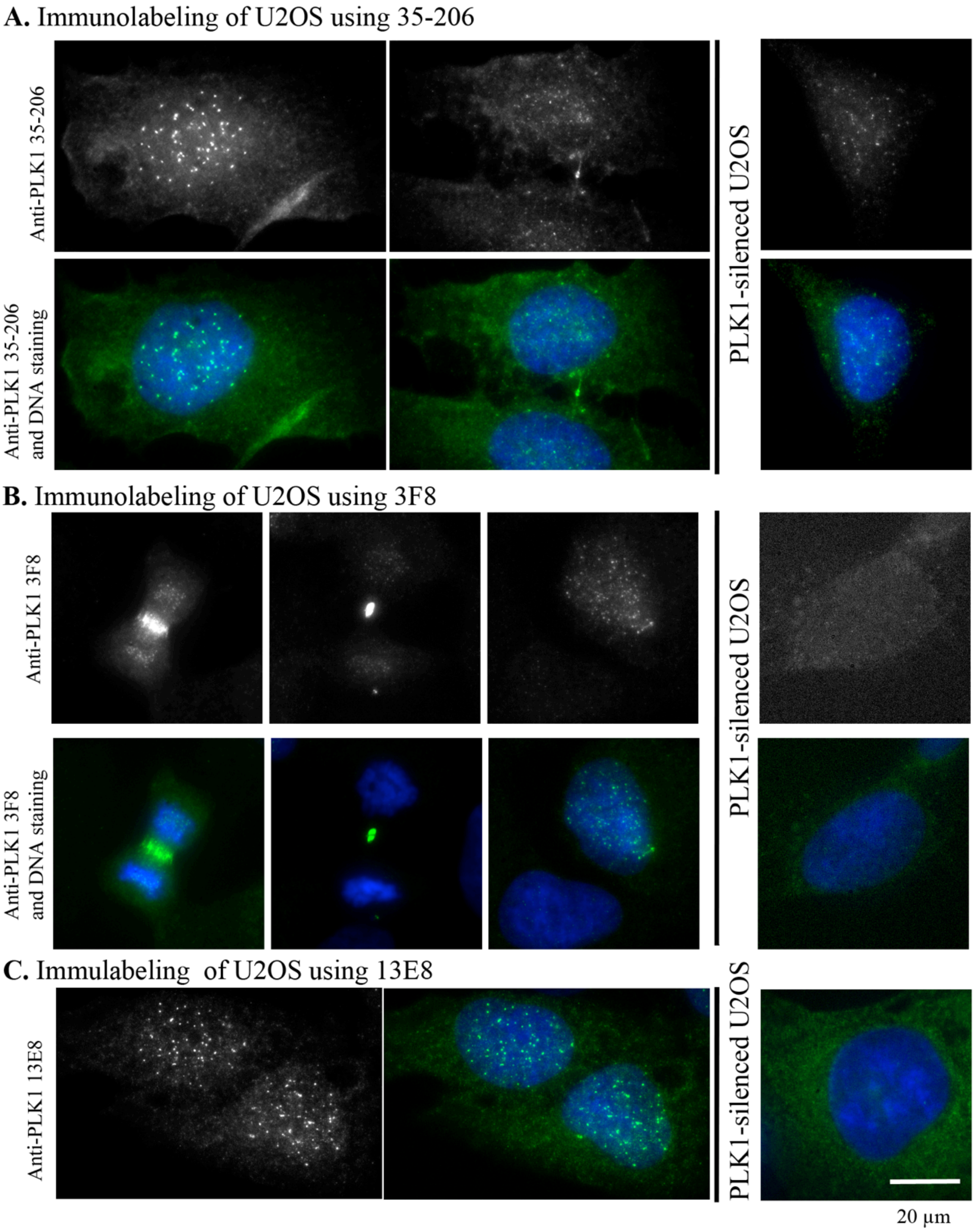

Figure S6. Classical immunofluorescence detection of PLK1 in U2OS cells. Wild type U2OS or PLK1-silenced U2OS (24 h post electroporation) were fixed with 4% (w/v) paraformaldehyde, permeabilized with 0.1% (w/v) Triton X-100, and incubated with the indicated primary anti-PLK1 antibody, followed by an Alexa Fluor 488–conjugated anti-mouse secondary antibody. Nuclei were stained with Hoechst 33242. Signals corresponding to PLK1 and DNA are shown in the green and blue channels, respectively.

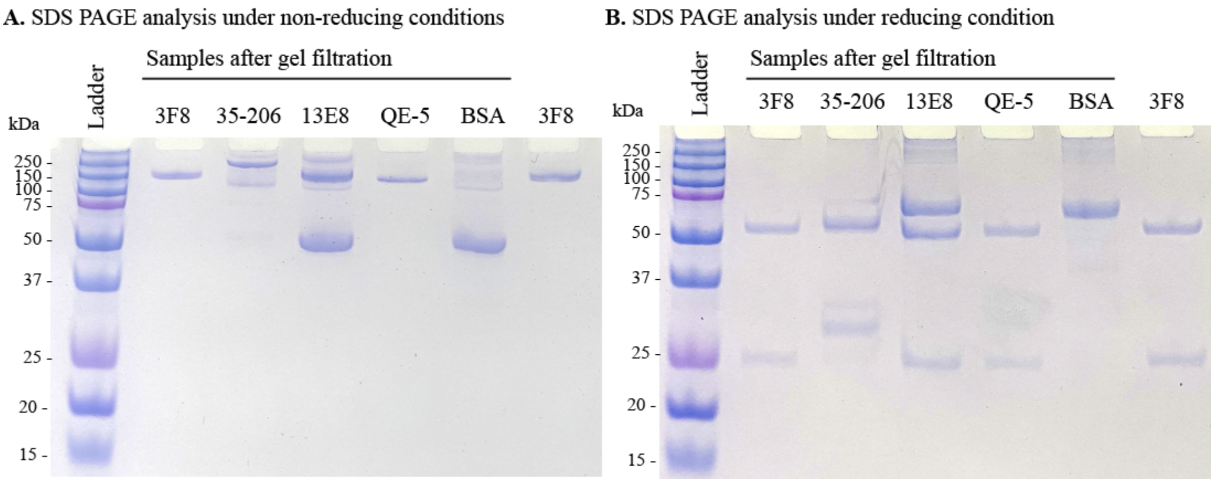

Figure S7. SDS–PAGE of the indicated mAbs under non-reducing (A) and reducing (B) conditions. Proteins (2 µg per lane) were stained with Coomassie Blue; BSA, bovine serum albumin. The 3F8 sample in the last lane is prior to gel-filtration. Under non-reducing conditions, 3F8, 13E8, and QE-5 migrate at ~150 kDa; 13E8 shows an extra band above 50 kDa (BSA); 35-206 migrates at ~250 kDa. Under reducing conditions, heavy (~50 kDa) and light (~25 kDa) chains appear for 3F8, 13E8, and QE-5, while 35-206 shows slightly higher bands (~55 kDa and ~30 kDa), likely from post-translational modifications or anomalous SDS binding.

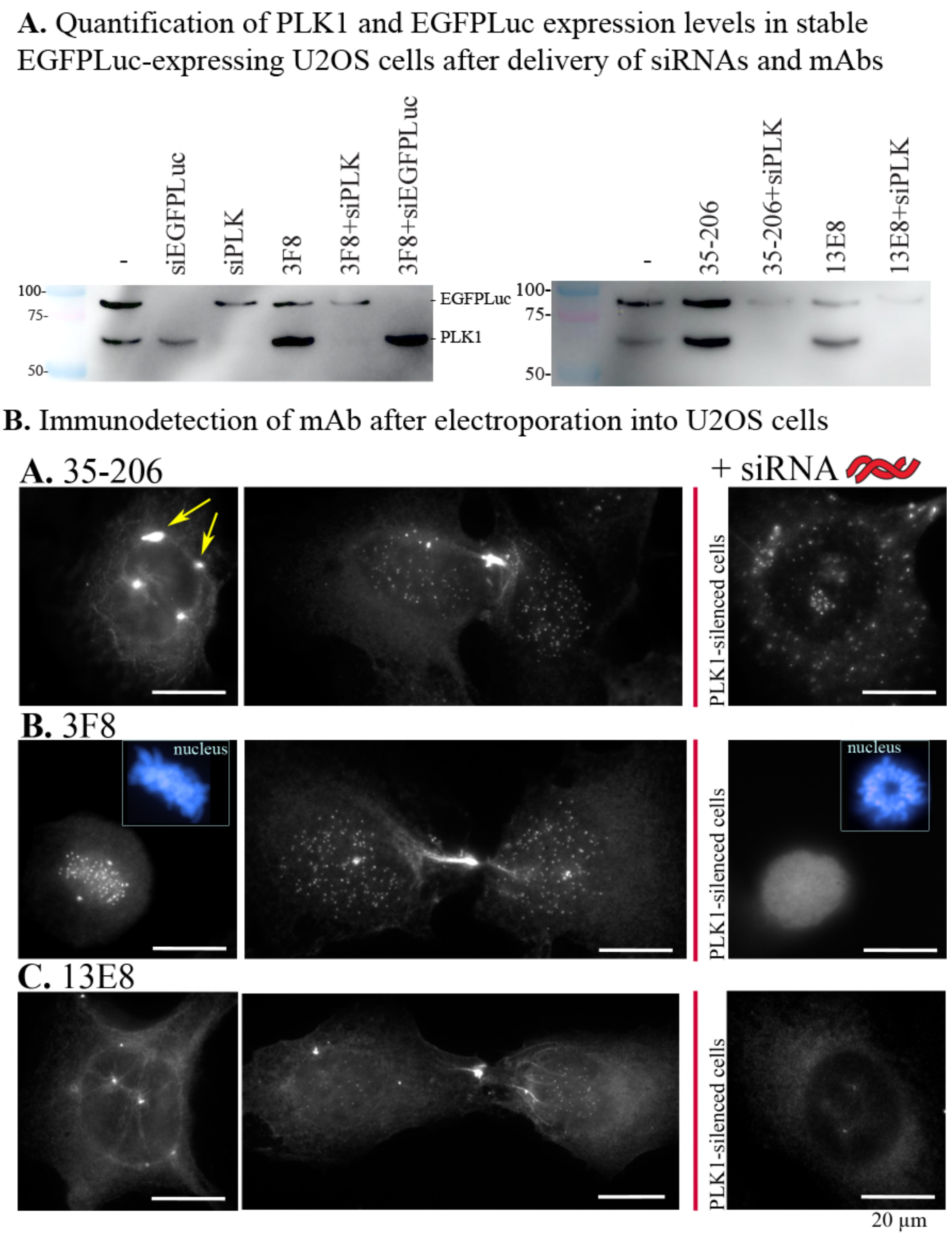

Figure S8. A. Quantification of siRNA-mediated gene silencing in EFGPLuc-U2OS cell line using electroporation to transfer siRNAs and antibodies. **B.** Immunodetection of the indicated anti-PLK1 monoclonal antibody 24 h after electroporation into wild-type U2OS cells. PLK1 expression was silenced by co-electroporation of the monoclonal antibody with PLK1-specific siRNA. Cells were fixed with 4% paraformaldehyde, permeabilized with 0.1% Triton X-100, and the monoclonal antibodies were detected using Alexa Fluor 488–conjugated secondary antibodies. Fluorescence images correspond to signals acquired in the green channel (mAb). Inset images are signals in the bleu channel (DNA staining). Yellow arrows point to apparently PLK1-unrelated detection. Electroporation conditions: 1.5 µM monoclonal antibody, 2 µM siRNA, 517 $V \cdot cm^{-1}$, 3 pulses of 10 ms.

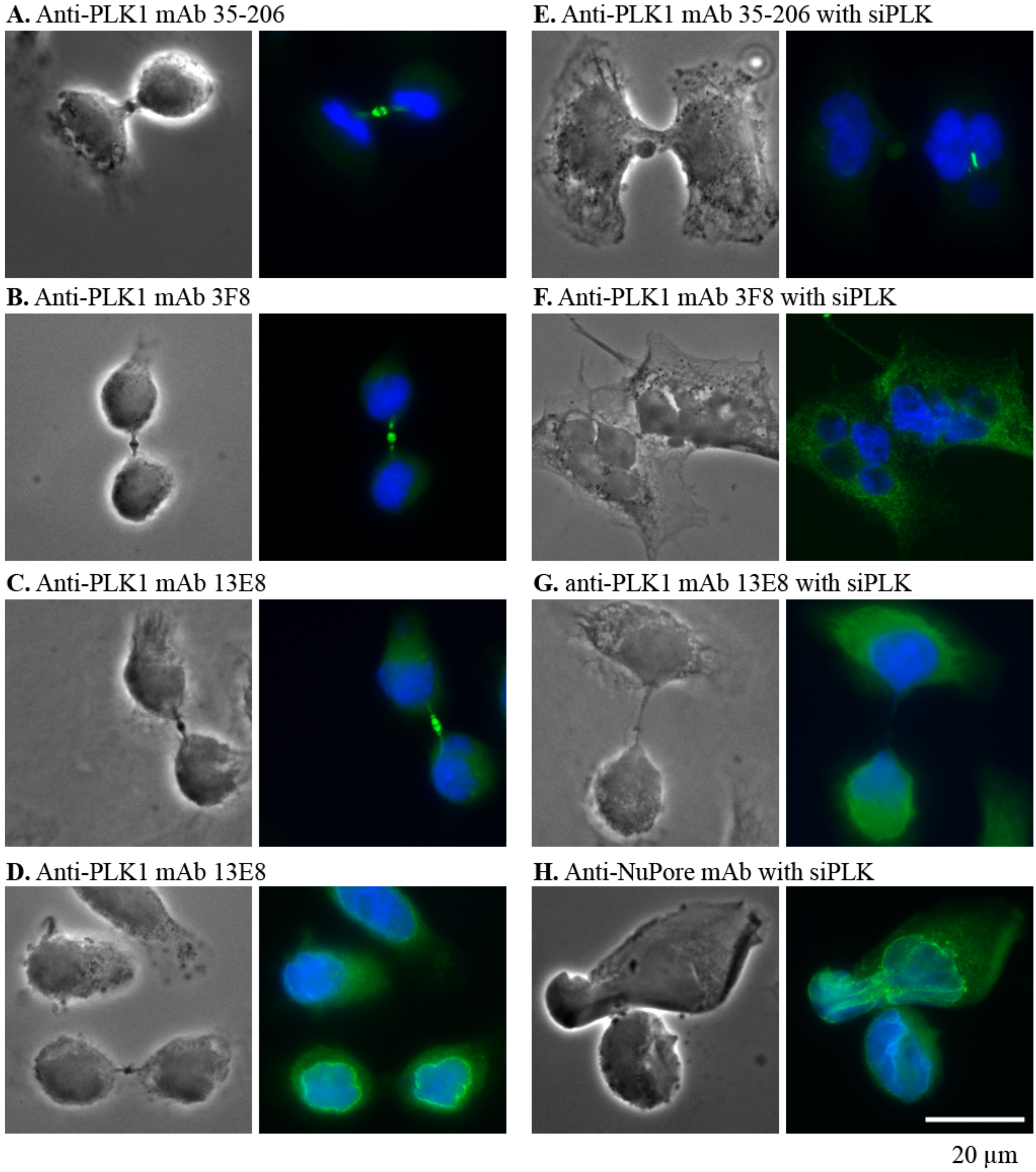

Figure S9. Immunodetection of the indicated monoclonal antibody 24 h after electroporation into HeLa cells. PLK1 expression was silenced by co-electroporation of the monoclonal antibody with PLK1-specific siRNA. Cells were fixed with 4% paraformaldehyde, permeabilized with 0.1% Triton X-100, and the monoclonal antibodies were detected using Alexa Fluor 488–conjugated secondary antibodies. Fluorescence images correspond to merged signals acquired in the green channel (mAb) and bleu channel (DNA). Images were selected to show the cytokinesis. Electroporation conditions: 1.5 μM monoclonal antibody, 2 μM siRNA, 517 $V \cdot cm^{-1}$, 3 pulses of 10 ms.

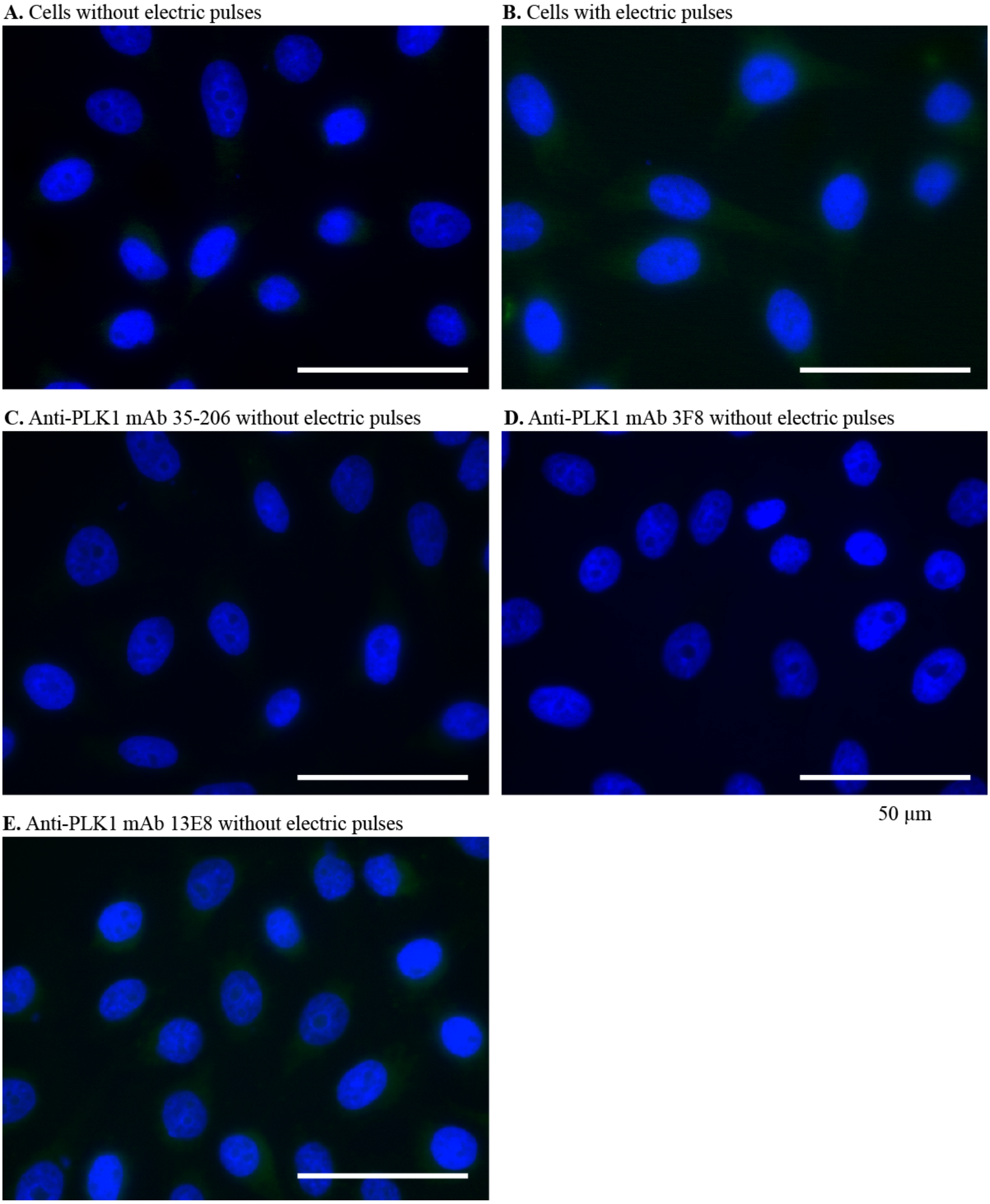

Figure S10. Detection of mAbs 18 h after incubation with HeLa cells without application of electric pulses. 1.1×10^5 cells in 10 µL PBS containing 1.5 µM mAb were diluted in medium and cultured for 18 h. Cells were then fixed with 4% PFA, permeabilized with Triton X-100, stained with Hoechst 33242, and mAbs were detected using Alexa Fluor 488–labeled secondary anti-mouse antibody. Green (detection of mAb) and blue (detection of nuclei) channels were merged. No accumulation of mAbs in intracellular vesicles was observed, and fluorescence in the green channel was extremely weak.

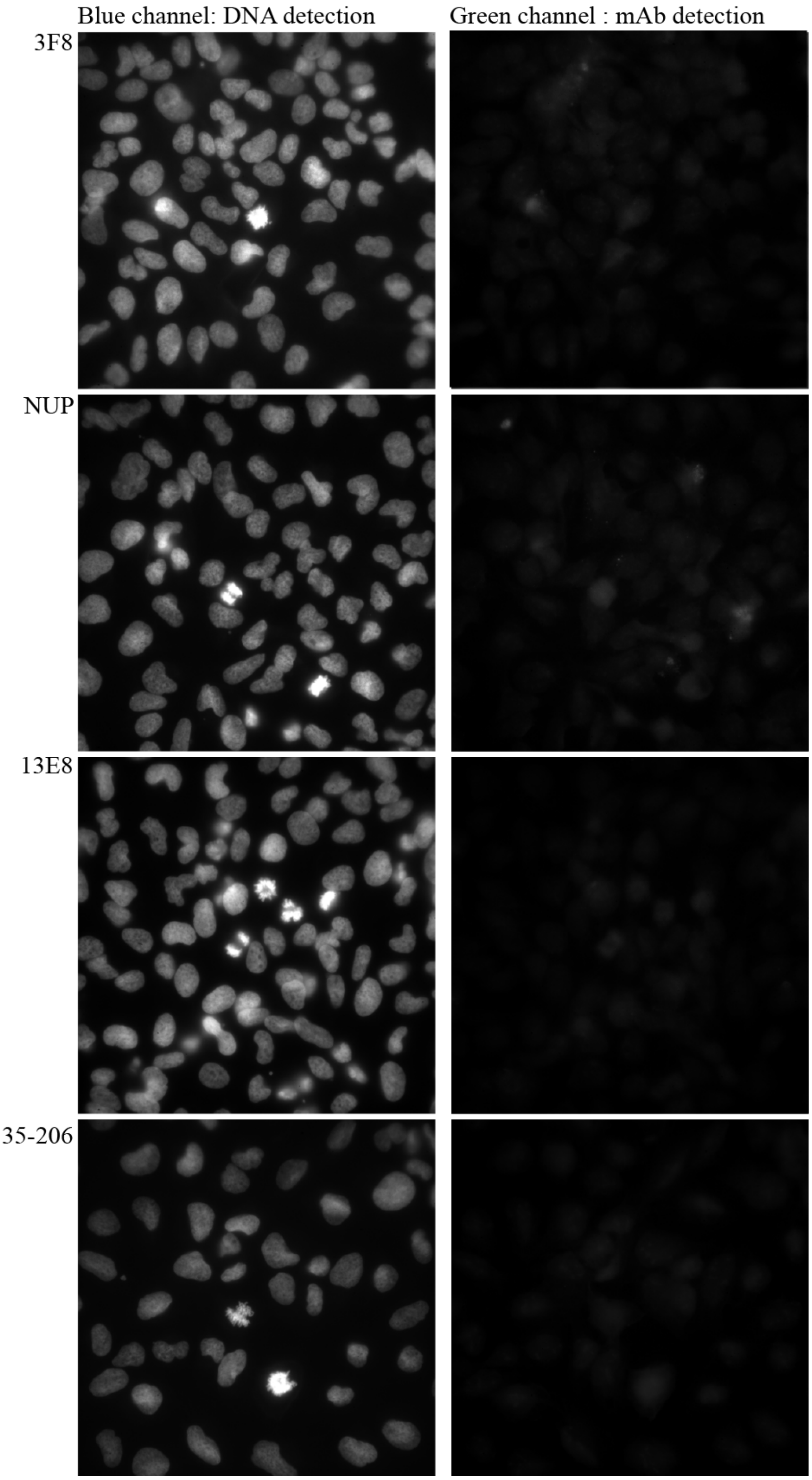

Figure S11. Detection of mAbs 18 h after incubation with U2OS cells without application of electric pulses. 1.1×10^5 cells in 10 µL PBS containing 1.5 µM mAb were diluted in medium and cultured for 18 h. Cells were then fixed with 4% PFA, permeabilized with Triton X-100, stained with Hoechst 33242, and mAbs were detected using Alexa Fluor 488–labeled secondary anti-mouse antibody. Fluorescence in the green channel was extremely weak. No accumulation of mAbs in intracellular vesicles was observed.

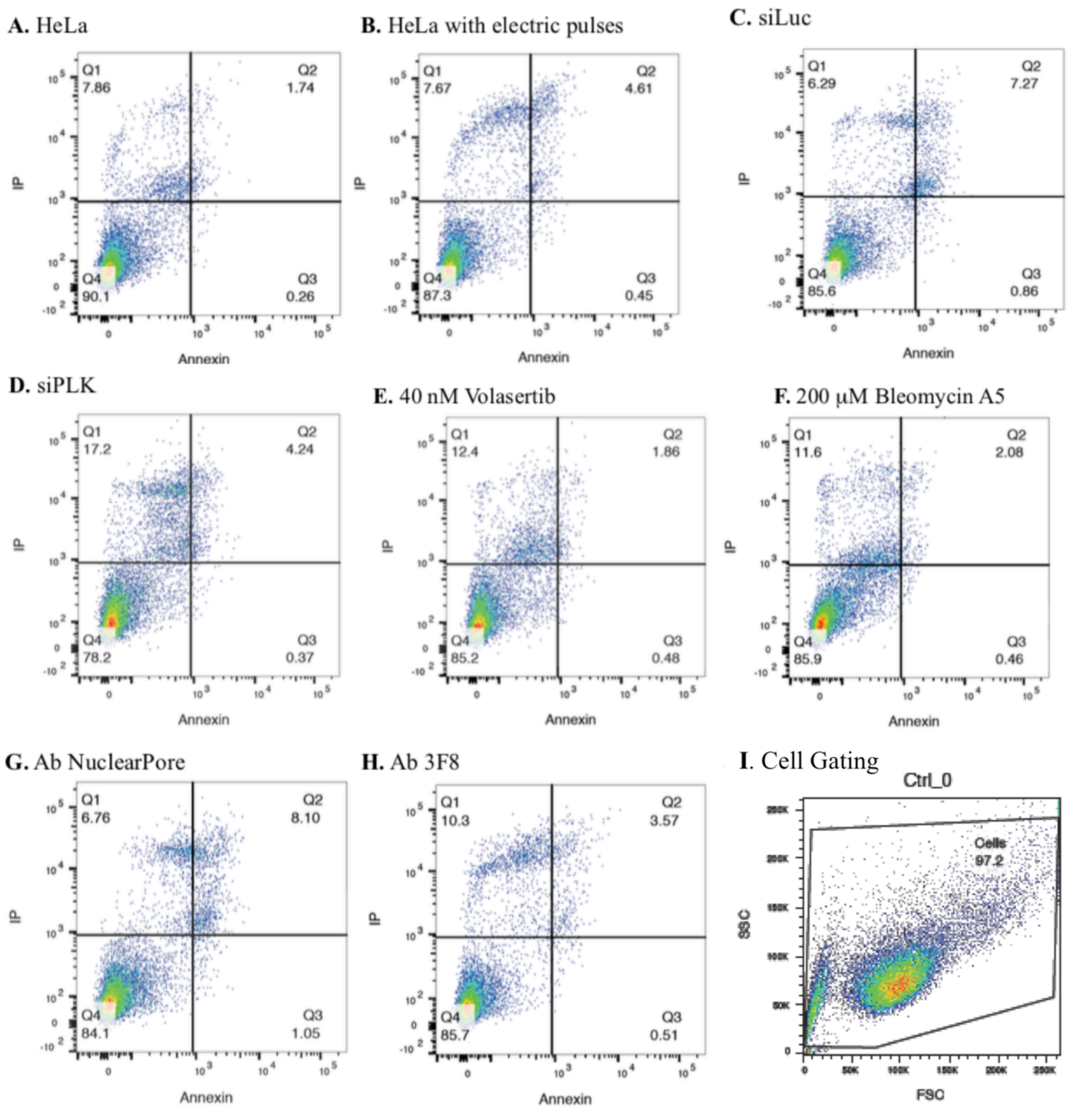

Figure S12. Cell death and apoptosis were evaluated by flow cytometry using Annexin V and propidium iodide 24 h after addition of the indicated compound with or without electroporation. Panels **A**, **E**, and **F**: no electroporation. Panels **B**, **C**, **D**, **G**, and **H**: electroporation. A total of 10,000 events were acquired per experience. Data were collected with minimal gating (see panel **I**). Condition : 3 µM monoclonal antibody, 517 $V{\cdot}cm^{-1}$, 3 pulses of 10 ms.

A. HeLa – siLuc **R/G : 0.4**

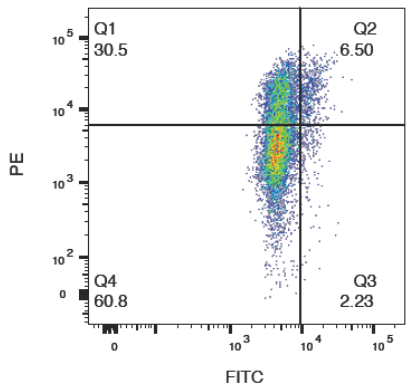

B. 200 μM Bleomycin A5 **R/G : 1.**

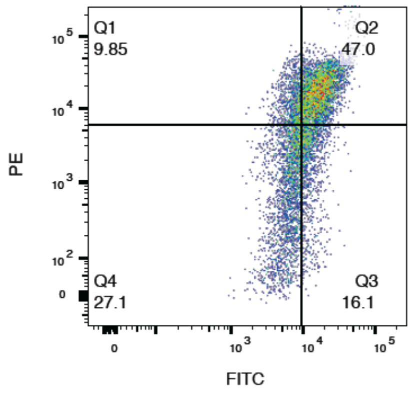

C. siPLK **R/G : 3.75**

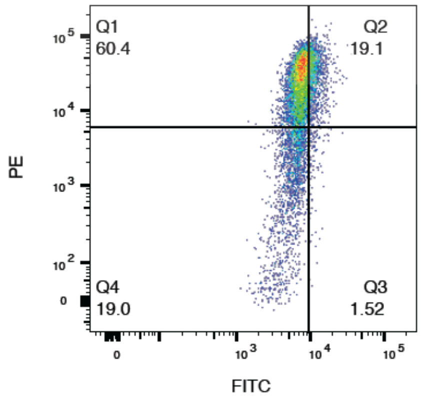

D. 40 nM Volasertib **R/G : 3.3**

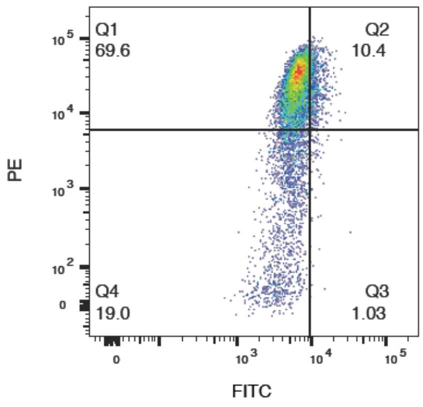

E. anti-PLK 3F8
2 main populations **R/G: 0.35 (60%) and 2.5 (40%)**

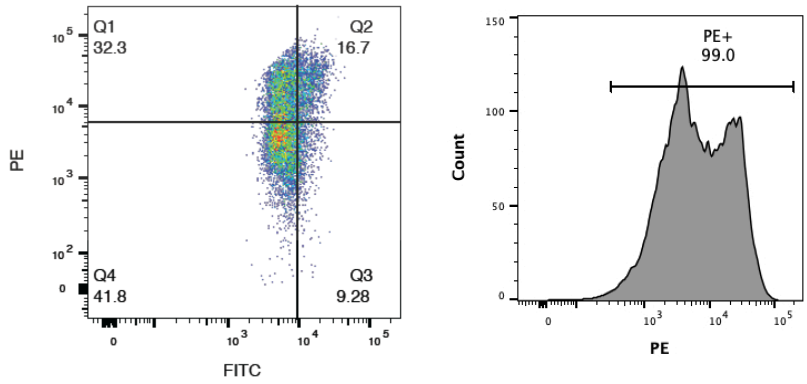

Figure S13. Evaluation of mitochondrial membrane potential by flow cytometry using JC-10 24 h after addition of the indicated compound. Panels **B** and **D**: no electroporation. Panels **A**, **C**, and **E**: electroporation. A total of 10,000 events were acquired per sample. Data were collected with minimal gating. The red (R) to green (G) fluorescence ratio (R/G) was used to monitor mitochondrial membrane polarization. An increased R/G ratio is indicative of mitochondrial damage. Condition: a total of 10,000 events were acquired per experience. Data were collected with minimal gating. 3 μM monoclonal antibody, 517 V·cm^{-1}, 3 pulses of 10 ms.

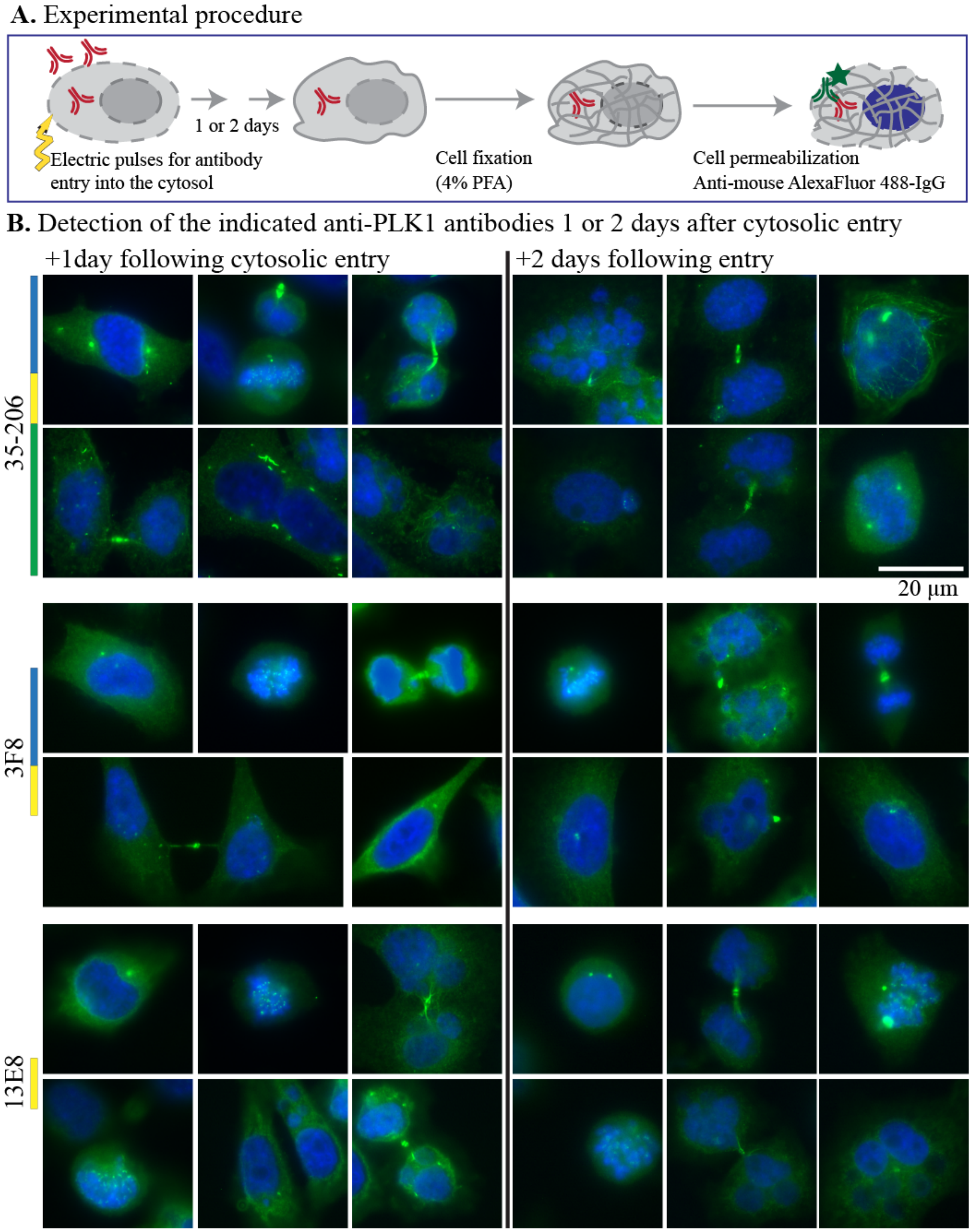

Figure S14. Immunodetection of the indicated anti-PLK1 monoclonal antibody 24 and 48 h after electroporation into HeLa cells. Cells were fixed with 4% paraformaldehyde, permeabilized with 0.1% Triton X-100 and stained with Hoechst 33242. The monoclonal antibodies were detected using Alexa Fluor 488–conjugated secondary antibodies. Fluorescence images correspond to merged signals acquired in the green channel (mAb) and blue channel (DNA). Electroporation conditions: 3 µM monoclonal antibody, 517 $V \cdot cm^{-1}$, 3 pulses of 10 ms.

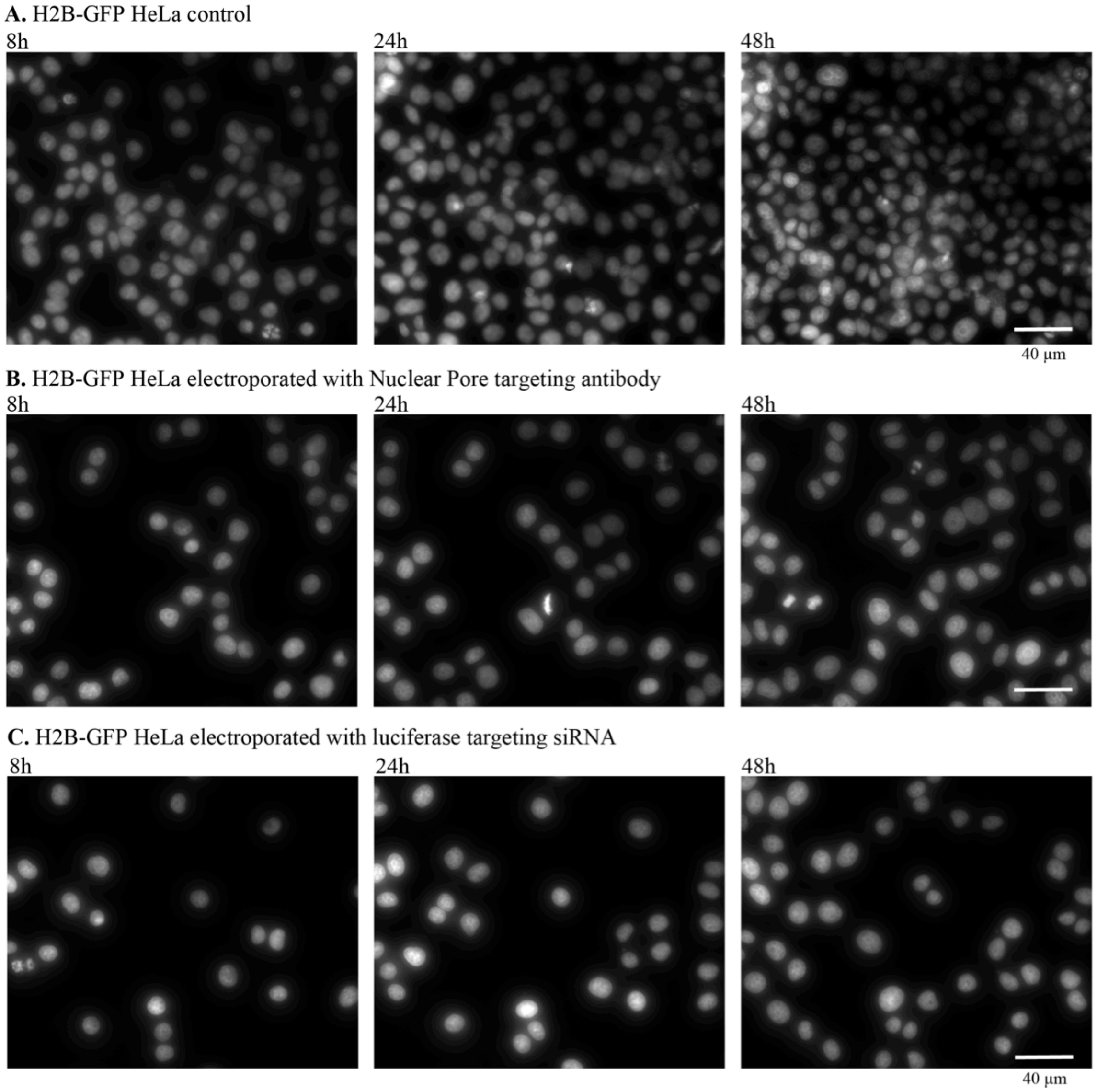

Figure S15. Images extracted from time-lapse movies of nuclei from H2B-GFP HeLa cells treated as indicated and at the specified time points. Nuclei were counted and classified according to cell cycle status (interphase or mitosis) or as dead cells.

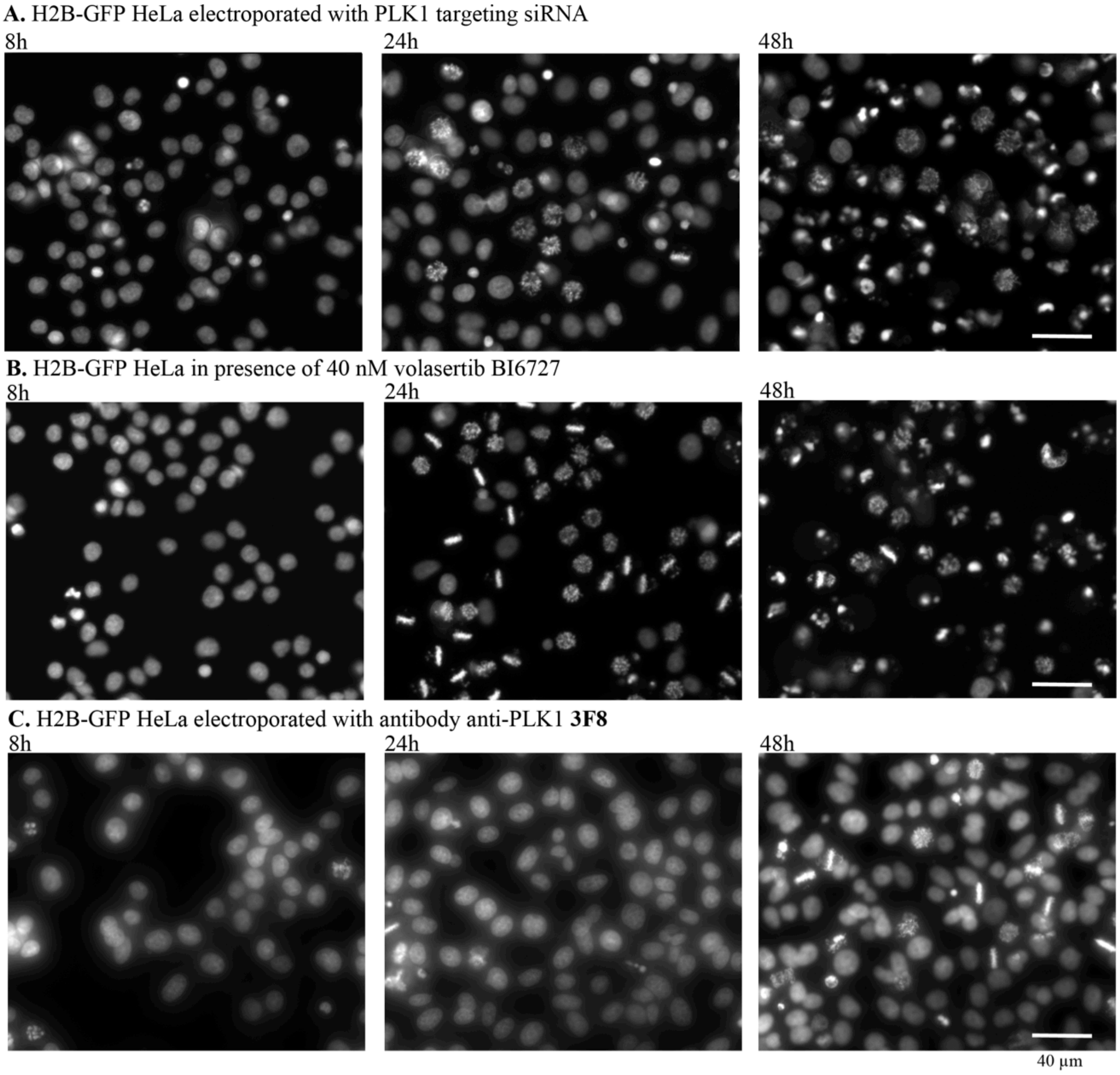

Figure S16. Images extracted from time-lapse movies of nuclei from H2B-GFP HeLa cells treated as indicated and at the specified time points. Nuclei were counted and classified according to cell cycle status (interphase or mitosis) or as dead cells.

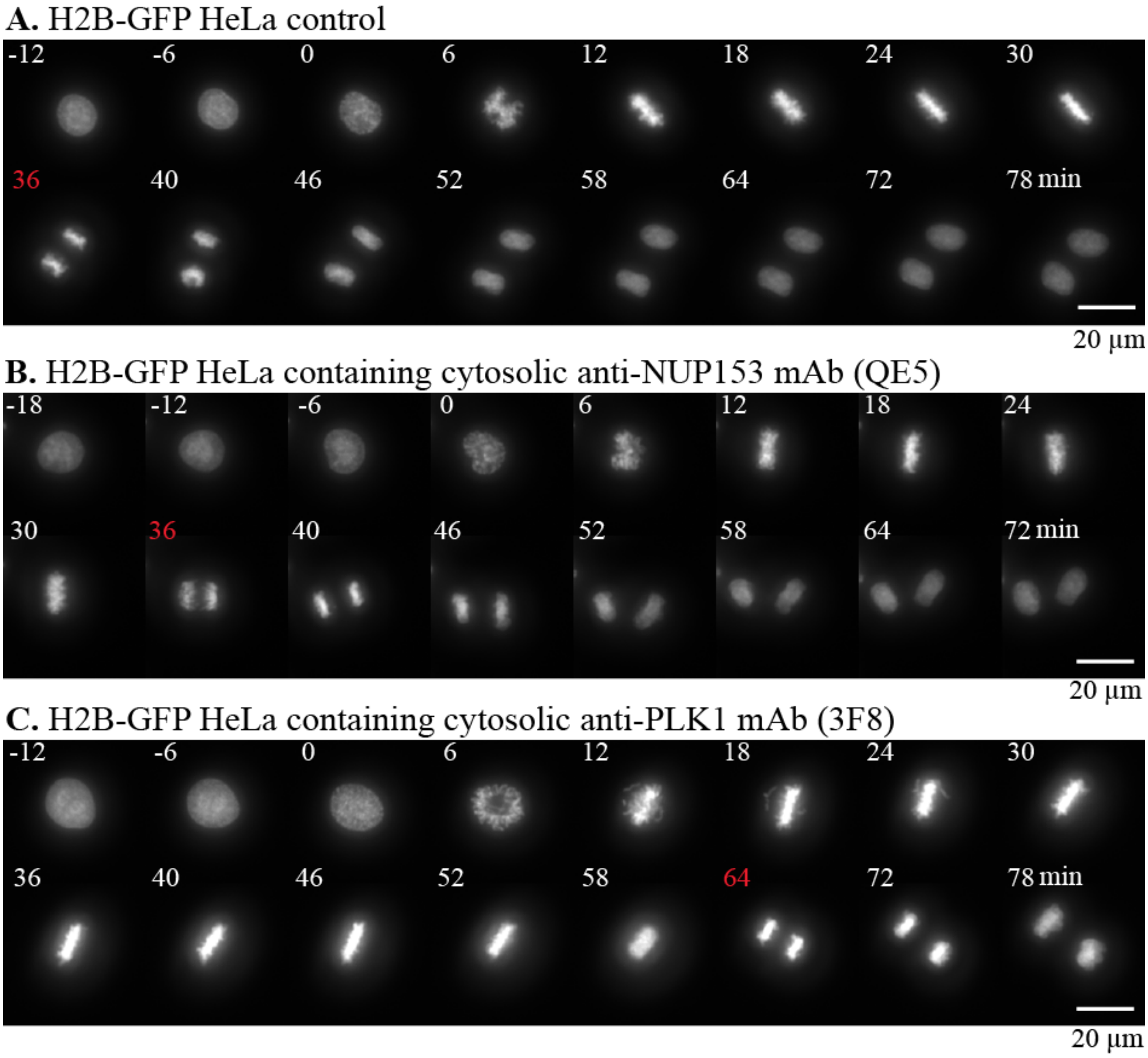

Figure S17. Impact of cytosolic mAbs after delivery into living H2B-GFP HeLa cells. **A.** Control. **B and C.** The indicated mAb (3 µM) were delivered into synchronized HeLa cells; time-lapse imaging shows increased duration of mitosis in presence of 3F8 mAb.

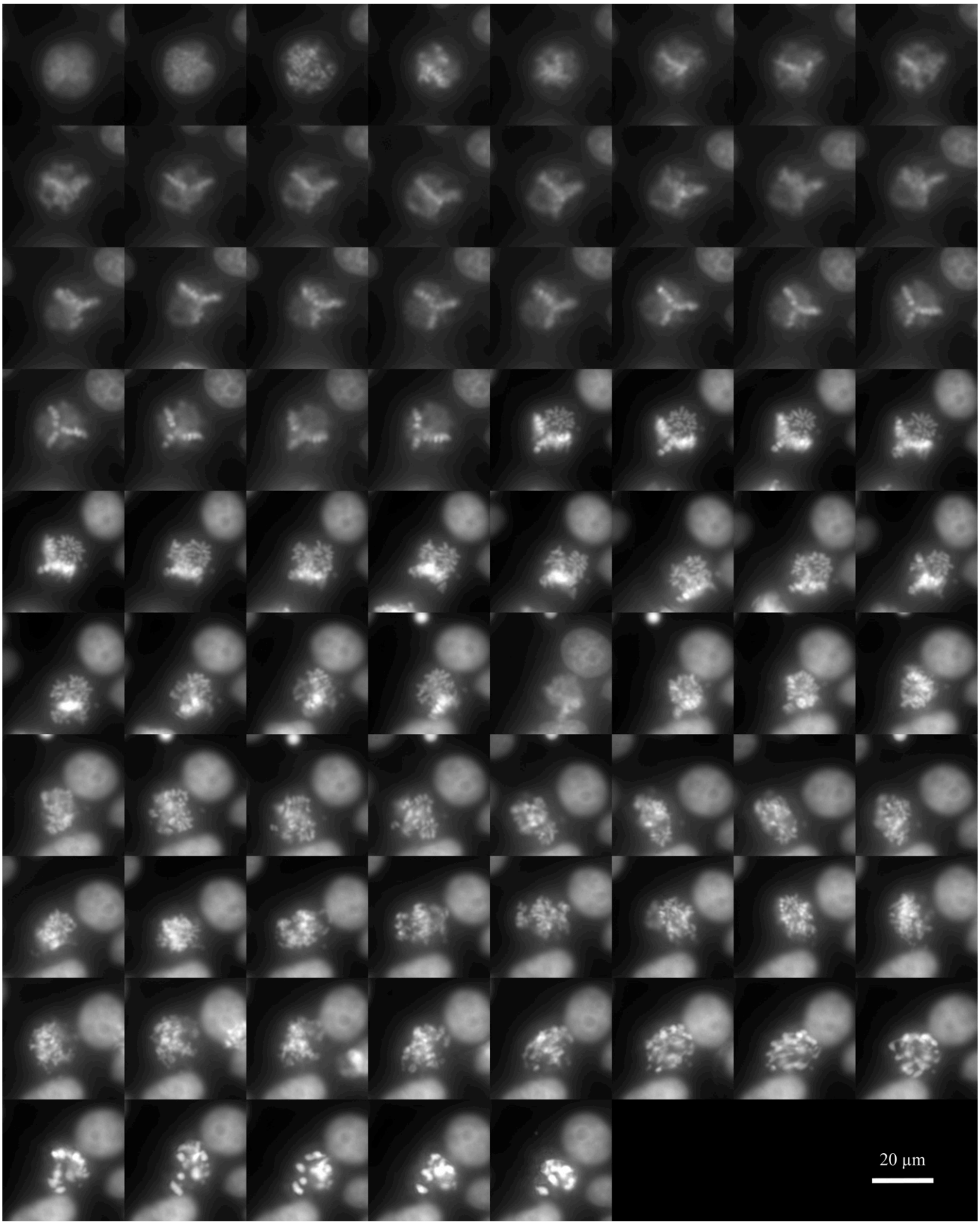

Figure S18. Impact of 3F8 mAbs after delivery into living H2B-GFP HeLa cells. Synchronized cells were electroporated with 3 µM 3F8. Time lapse imaging of cell nuclei was then performed Montage shows the nucleus of a cell undergoing extended mitosis duration (more than 640 min) and then fragmented, in a sign of apoptosis. Images were acquired every 10 min. Electroporation conditions: 517 V·cm^{-1}, 3 pulses of 10 ms (n = 10^5 cells).

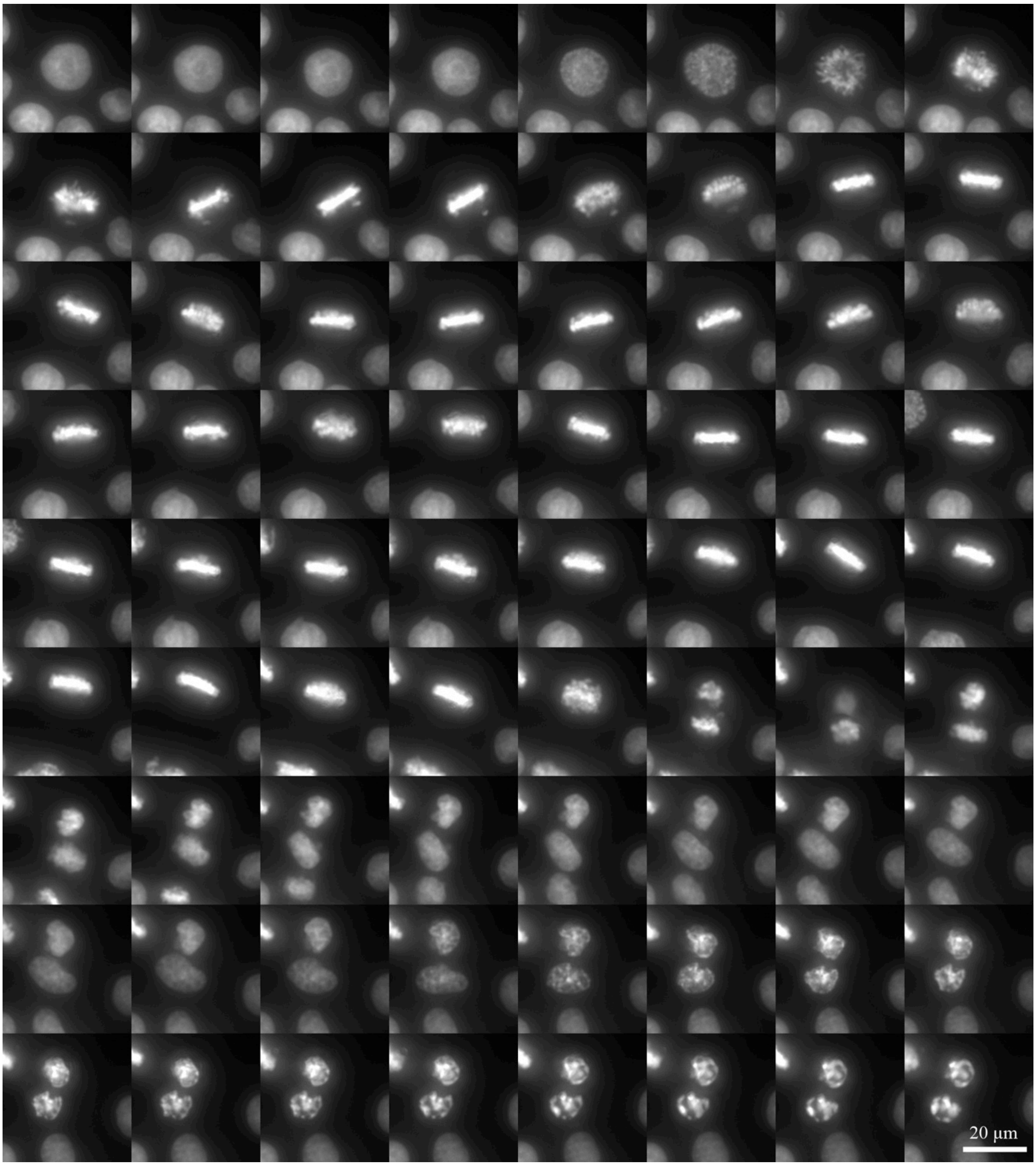

Figure S19. Impact of 3F8 mAbs after delivery into living H2B-GFP HeLa cells. Synchronized cells were electroporated with 3 µM 3F8. Time lapse imaging of cell nuclei was then performed Montage shows the nucleus of a cell undergoing extended mitosis duration (380 min), divided and then fragmented. Images were acquired every 10 min. Electroporation condition: 517 V·cm^{-1}, 3 pulses of 10 ms (n = 10^5 cells).

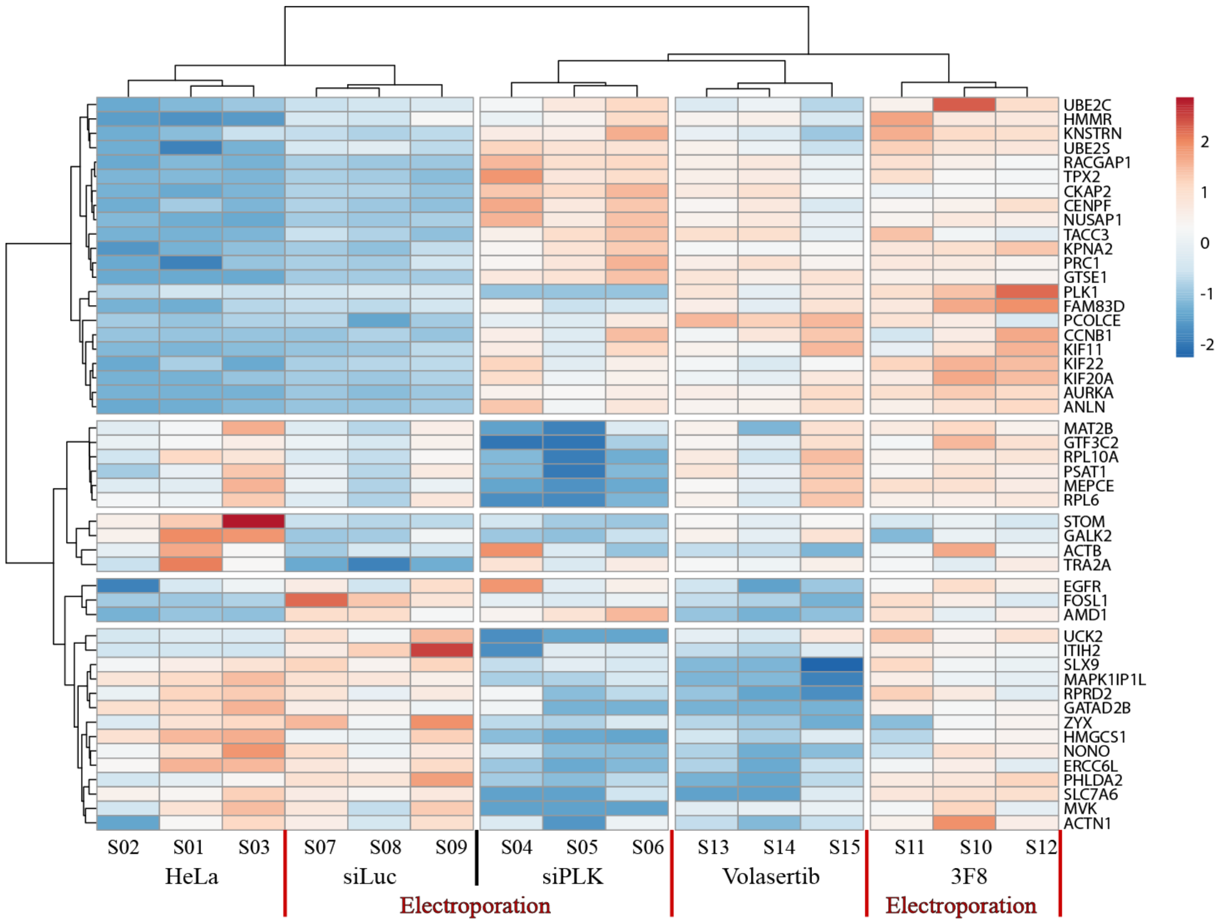

Figure S20. Euclidean hierarchical clustering of the 49 significant proteins identified in the proteomic analysis. Synchronized HeLa cells were electroporated with luciferase siRNA (siLuc, 2 µM), PLK1 siRNA (siPLK1, 2 µM), or the anti-PLK1 mAb 3F8 (3 µM). Control conditions included non-electroporated cells and cells treated with Volasertib (40 nM). Cells were analyzed 24 h after electroporation (517 V·cm^{-1}, 3 pulses of 10 ms). Samples treated with PLK1-targeting agents showed a cluster of 22 upregulated proteins (UBE2C → ANLN). Functional analysis using STRING (string-db.org) indicated that 20 of these 22 proteins are involved in spindle elongation and PLK–mediated processes. The two proteins not associated with these pathways were KNSTRN and PCOLCE.

A. UV-vis spectra of Alexa Fluor488-labeled mAb

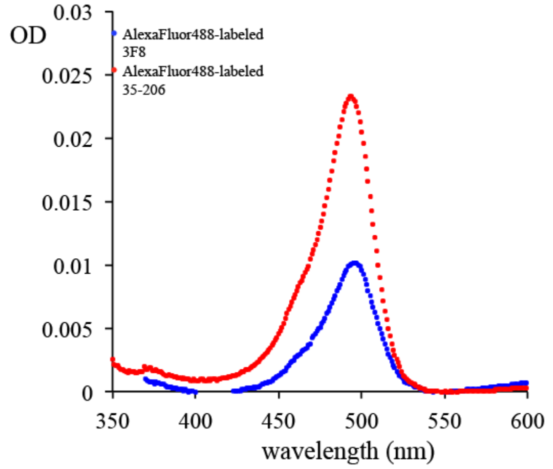

Absorption coefficient of Alexa Fluor 488
ε488 nm: 73000 M^{-1}.cm^{-1}

	Concentration (µM)		Labeling Ratio
	mAb	Alexa488	
mAb **3F8**	0.06	0.1212	**2.02**
mAb **35-206**	0.0666	0.28557	**4.28**

B. Calibration of the green channel with beads

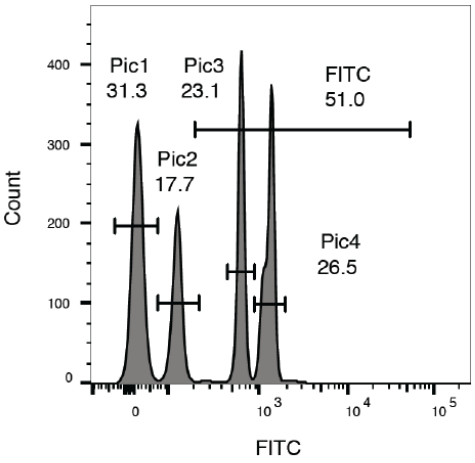

Median Bead 1: 22
Bead 2: 135
Bead 3: 649
Bead 4: 1347

C. Internalization of Alexa Fluor 488 conjugated 35-206 into HeLa

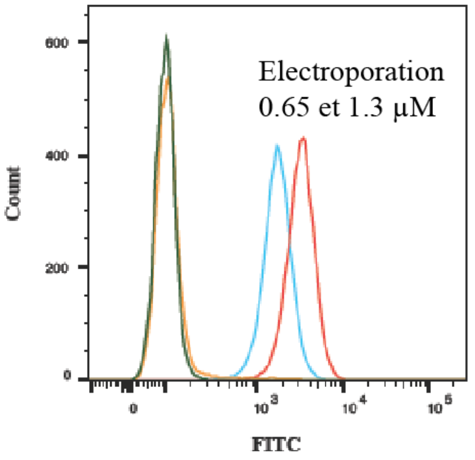

Sample Name	Count	Mean : FITC-A	Median : FITC-A
1_Cellules electro	9859	105	102
9_Cellules non electro 35206 C1	9911	204	110
4_Cellules electro 35206 C1	9765	3365	3227
5_Cellules electro 35206 C2	9396	1842	1740

Figure S21. Analysis of Alexa Fluor 488–conjugated antibodies. (A) UV–vis spectra of the conjugates after dilution. Protein concentrations were determined using a Bradford assay. (B) Calibration of the cytometer green fluorescence channel using calibrated Alexa Fluor beads (7 µm). (C) Flow cytometry analysis of the internalization of Alexa Fluor 488–labeled 35-206 into HeLa cells 5 h after electroporation at the indicated extracellular concentrations. Condition: 517 V·cm^{-1}, 3 pulses of 10 ms (n = 10^5 cells).

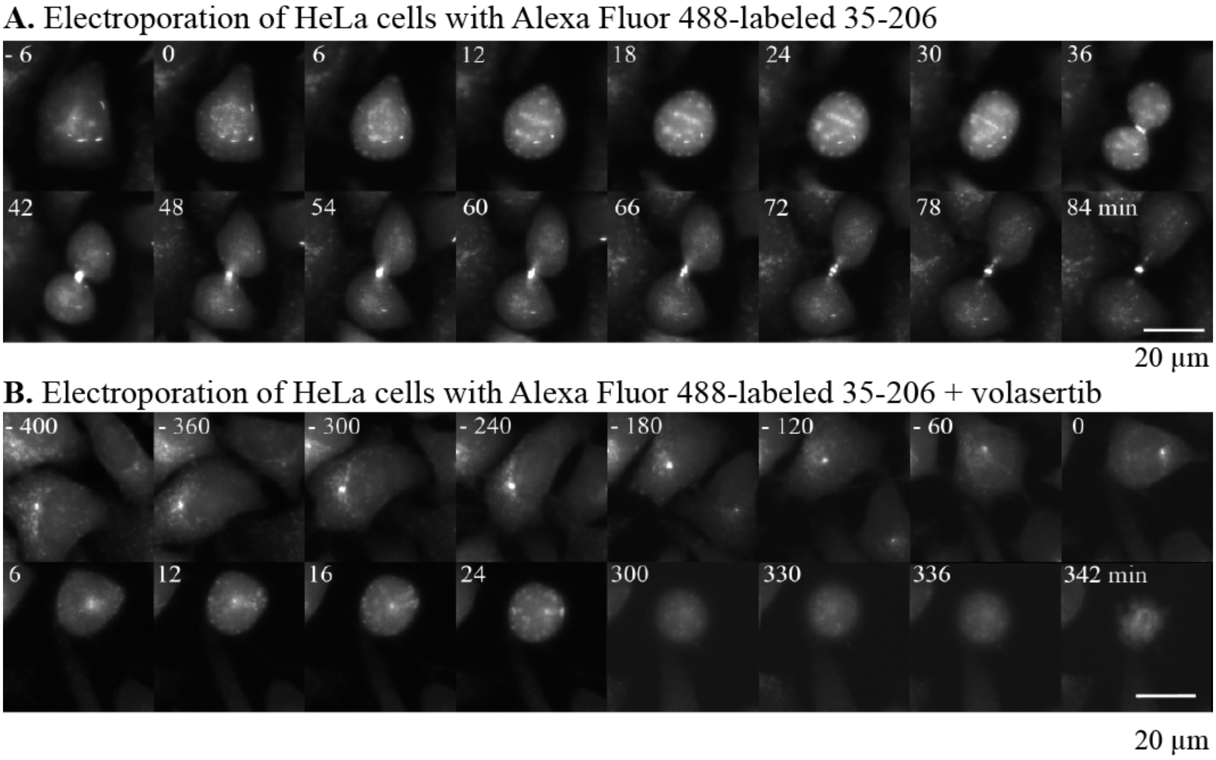

Figure S22. Live-cell fluorescence imaging of HeLa cells following electroporation with Alexa Fluor 488–labeled 35-206 alone (**A**) or co-treated with Volasertib (**B**). Cells were synchronized using a double thymidine block. Electroporation was performed upon release from DNA replication arrest. Condition: 3 μM antibody, 517 $V \cdot cm^{-1}$, 3 pulses of 10 ms (n = 10^5 cells). Movies were acquired starting 8 h after electroporation. Volasertib was added 4 h after electroporation to a final concentration of 40 nM. Note the presence of labeling apparently unrelated to PLK1 in the first images of montage **A**.

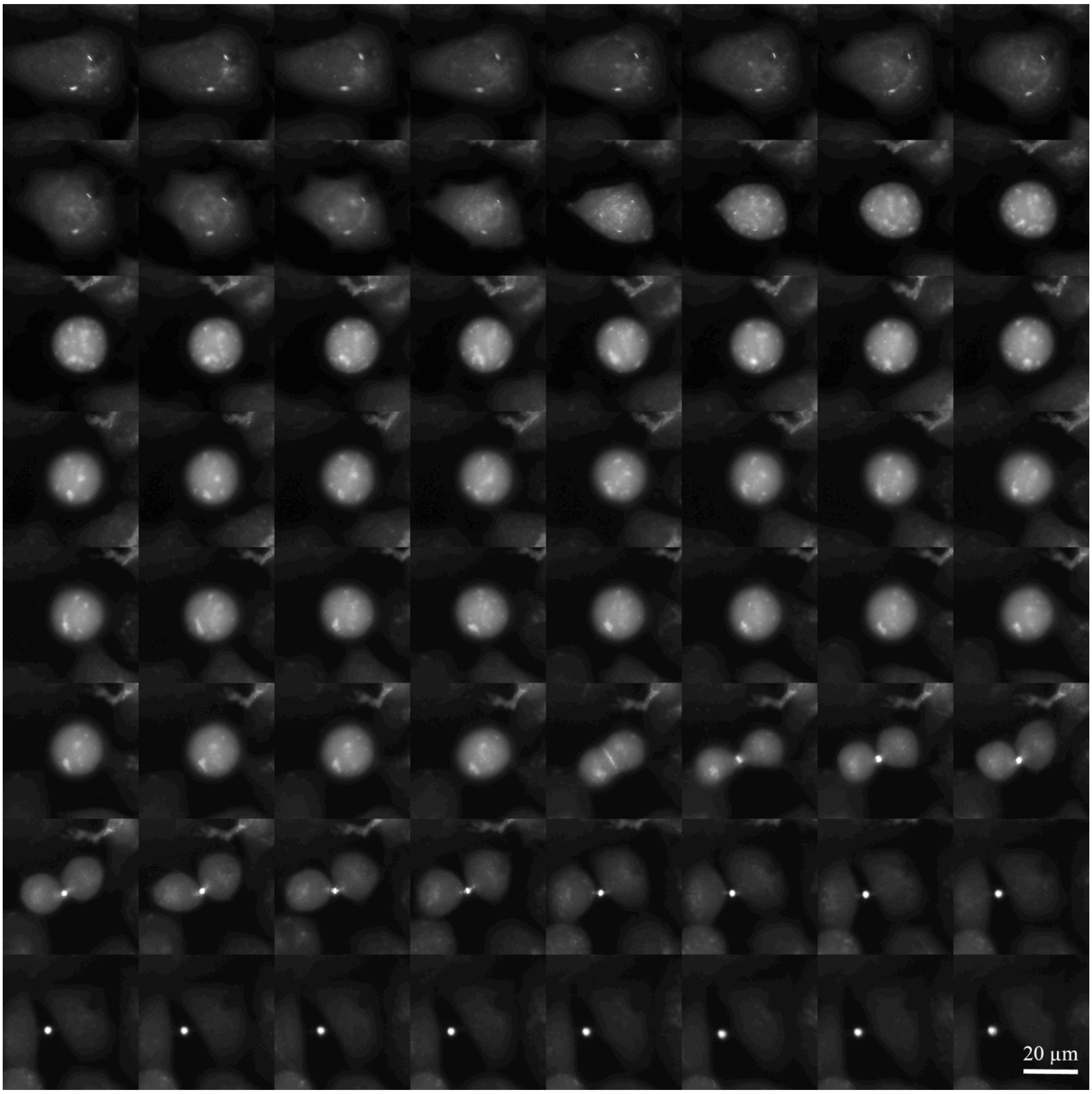

Figure S 23. Live-cell fluorescence imaging of HeLa cells following electroporation with Alexa Fluor 488–labeled 35-206. Cells were synchronized using a double thymidine block. Electroporation was performed upon release from DNA replication arrest. Condition: 3 μM antibody, 517 $V \cdot cm^{-1}$, 3 pulses of 10 ms ($n = 10^5$ cells). Images were acquired every 6 min starting 8 h after electroporation. Duration of mitosis: 216 min. Note the presence of labeling apparently unrelated to PLK1 in the first images.

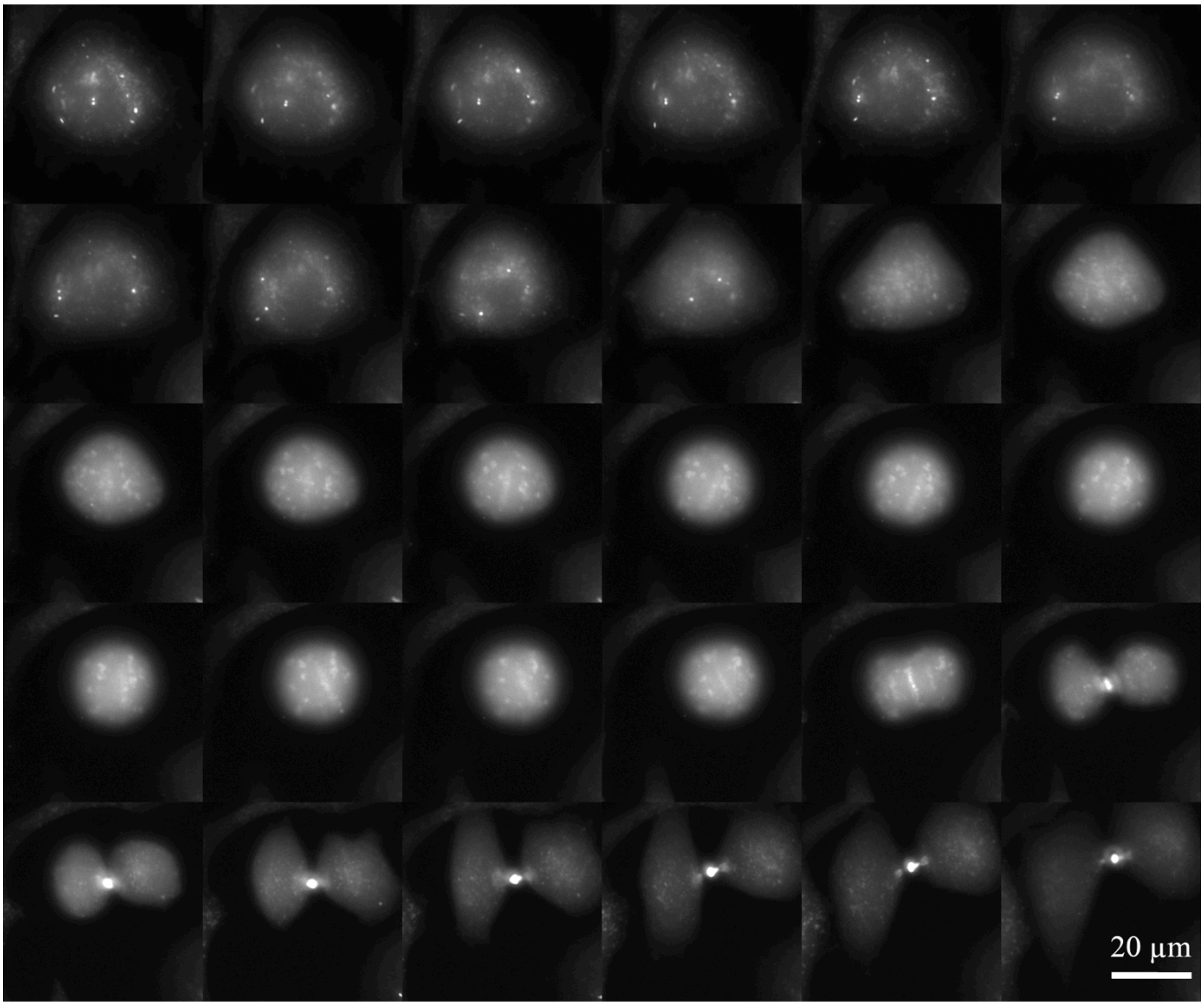

Figure S24. Live-cell fluorescence imaging of HeLa cells following electroporation with Alexa Fluor 488–labeled 35-206. Cells were synchronized using a double thymidine block. Electroporation was performed upon release from DNA replication arrest. Condition: 3 μM antibody, 517 $V{\cdot}cm^{-1}$, 3 pulses of 10 ms (n = 10^5 cells). Images were acquired every 6 min starting 8 h after electroporation. Duration of mitosis: 78 min. Note the presence of labeling apparently unrelated to PLK1.

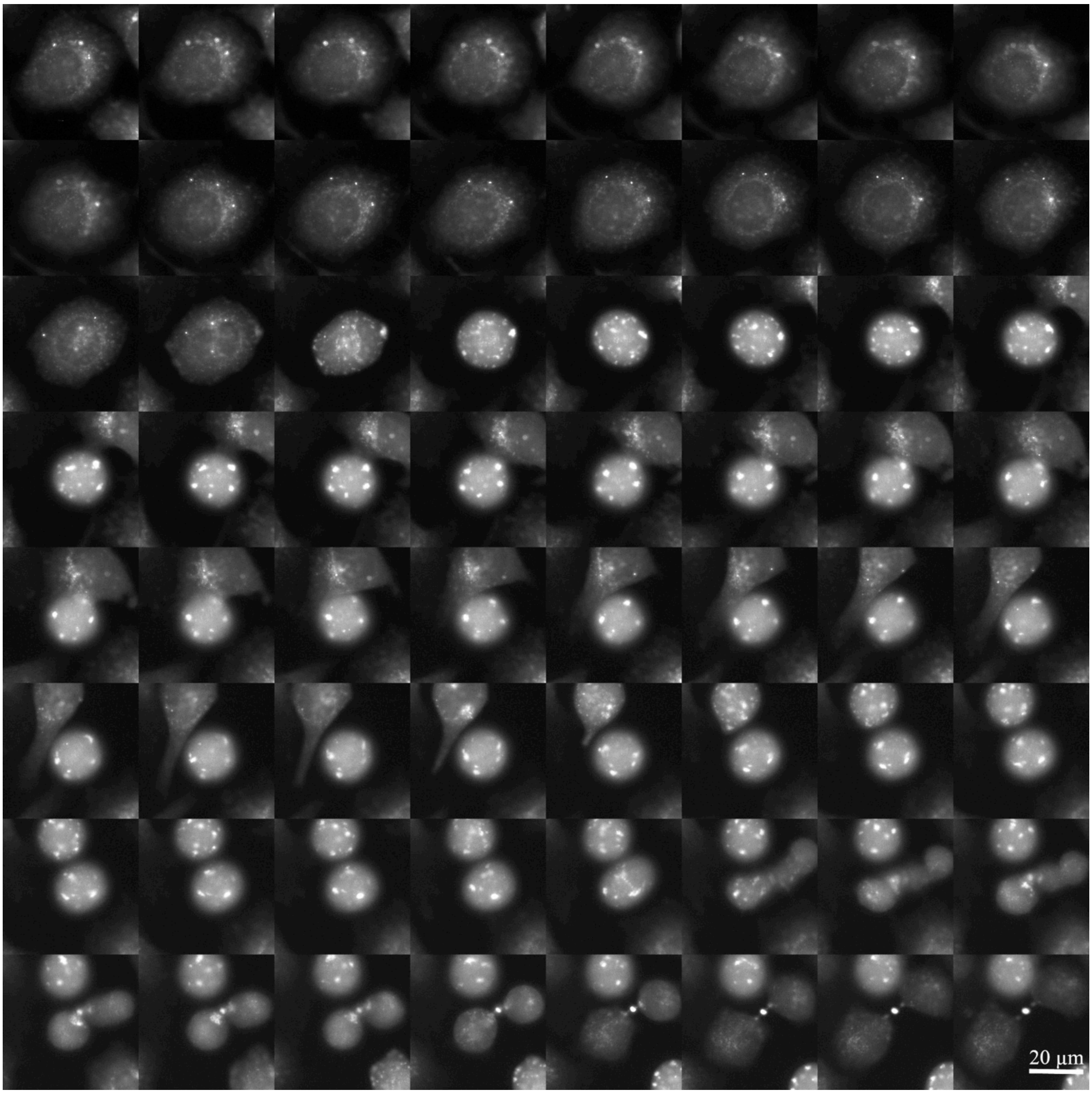

Figure S 25. Live-cell fluorescence imaging of HeLa cells following electroporation with Alexa Fluor 488–labeled 35-206. Cells were synchronized using a double thymidine block. Electroporation was performed upon release from DNA replication arrest. Condition: 3 µM antibody, 517 V·cm^{-1}, 3 pulses of 10 ms (n = 10^5 cells). Images were acquired every 6 min starting 8 h after electroporation. Duration of mitosis: 210 min. Note the presence of labeling apparently unrelated to PLK1.

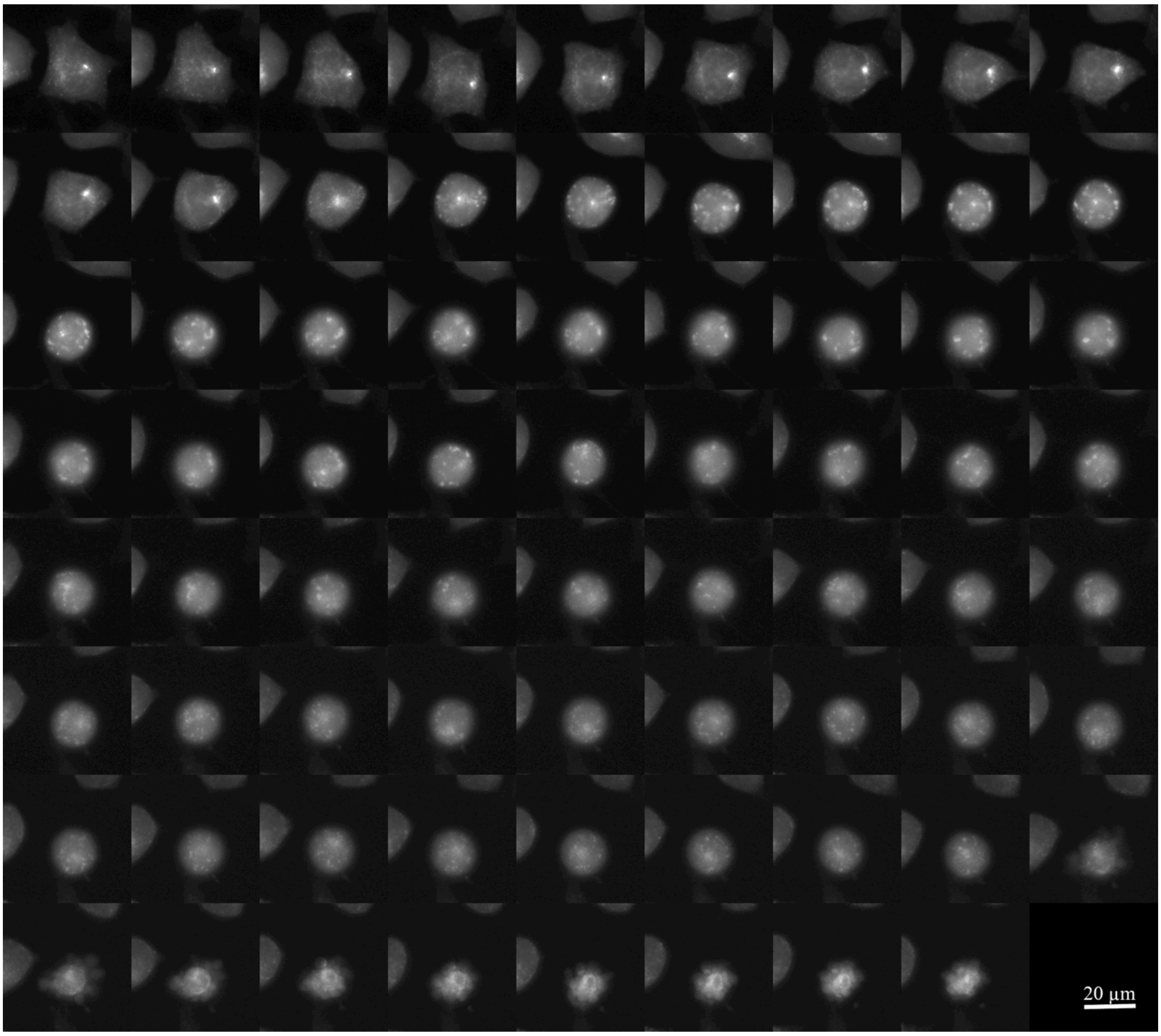

Figure S26. Live-cell fluorescence imaging of HeLa cells following electroporation with Alexa Fluor 488–labeled 35-206 and incubation with 40 nM volasertib. Cells were synchronized using a double thymidine block. Electroporation was performed upon release from DNA replication arrest. Condition: 3 μM antibody, 517 $V\cdot cm^{-1}$, 3 pulses of 10 ms (n = 10^5 cells). Images were acquired every each 6 min starting 8 h after electroporation. Duration of mitosis: 300 min before apoptosis.

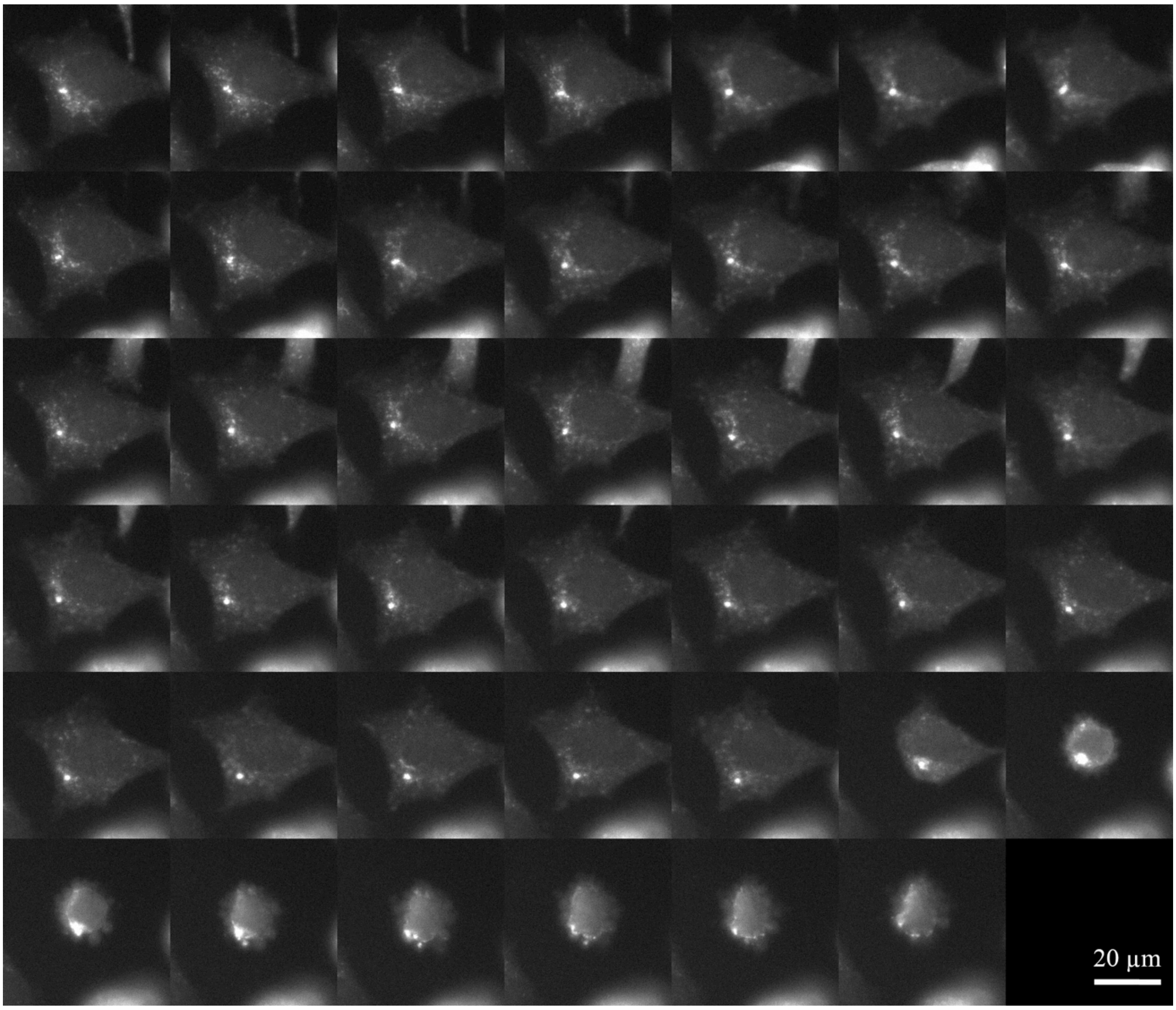

Figure S27. Live-cell fluorescence imaging of HeLa cells following electroporation with Alexa Fluor 488–labeled 35-206 and incubation with 40 nM volasertib. Cells were synchronized using a double thymidine block. Electroporation was performed upon release from DNA replication arrest. Condition: 3 μM antibody, 517 $V \cdot cm^{-1}$, 3 pulses of 10 ms (n = 10^5 cells). Images were acquired every each 6 min starting 8 h after electroporation. Apoptosis seems to occurs at G2/M transition.

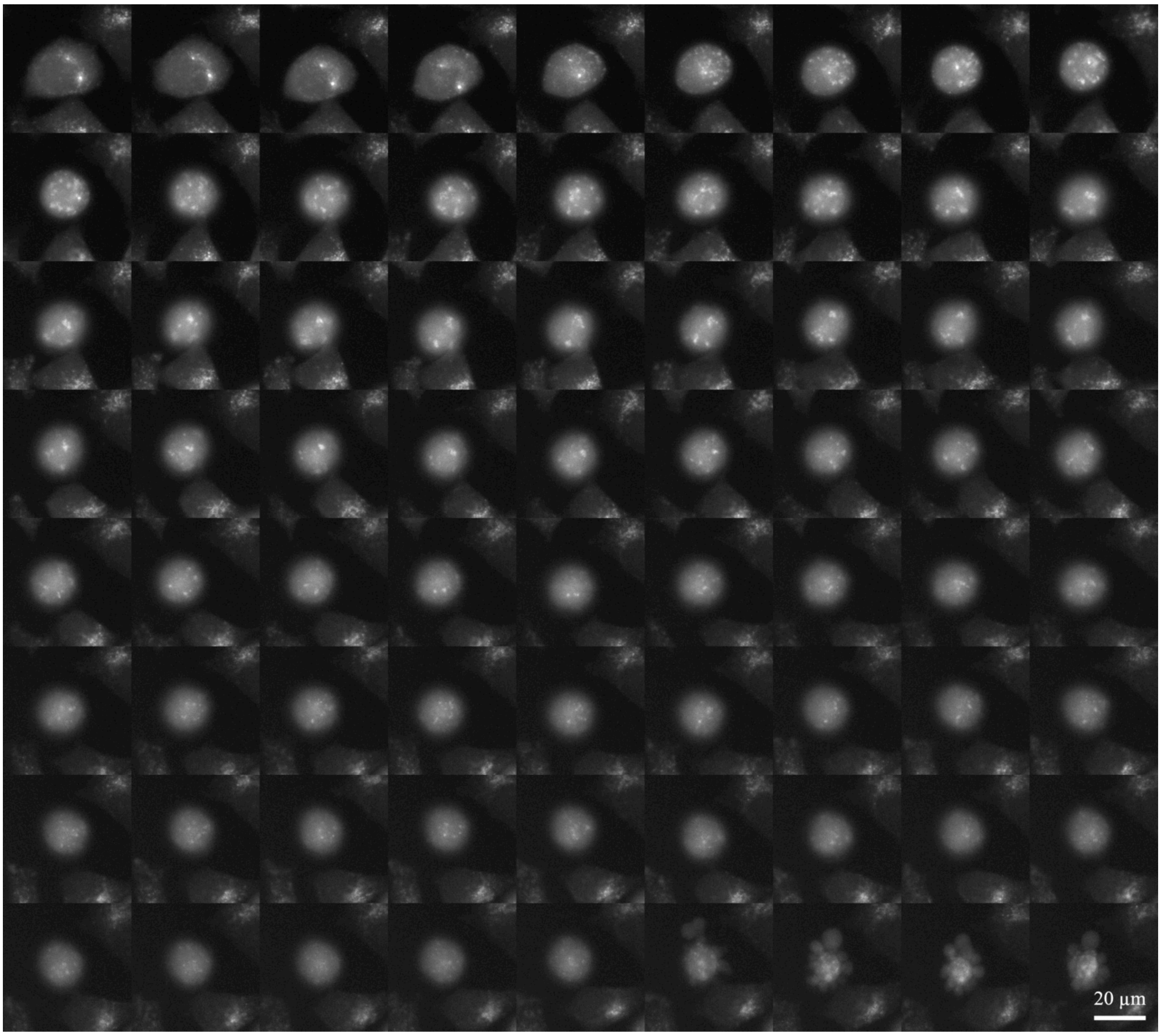

Figure S 28. Live-cell fluorescence imaging of HeLa cells following electroporation with Alexa Fluor 488–labeled 35-206 and incubation with 40 nM volasertib. Cells were synchronized using a double thymidine block. Electroporation was performed upon release from DNA replication arrest. Condition: 3 µM antibody, 517 $V \cdot cm^{-1}$, 3 pulses of 10 ms (n = 10^5 cells). Images were acquired every each 6 min starting 8 h after electroporation.

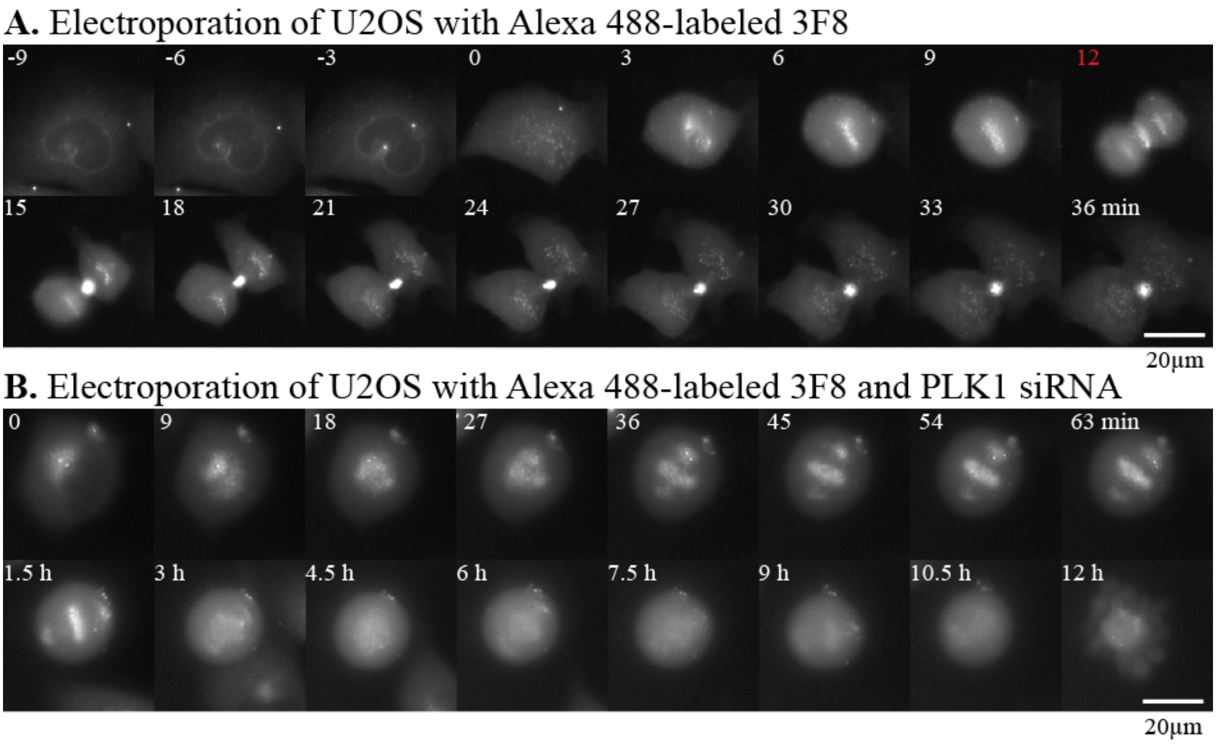

Figure S 29. Live-cell fluorescence imaging of U2OS cells following electroporation with Alexa Fluor 488–labeled 3F8. **A and B.** Cells were synchronized using a double thymidine block. Electroporation was performed upon release from DNA replication arrest. Condition: 3 μM antibody, 2 μM PLK1 siRNA, 517 $V{\cdot}cm^{-1}$, 3 pulses of 10 ms ($n = 10^5$ cells). Movies were acquired starting 8 h after electroporation
